# A *Pseudomonas aeruginosa* endolytic muramidase targets cell-wall peptidoglycan in bacterial competition

**DOI:** 10.1016/j.jbc.2025.110642

**Published:** 2025-08-28

**Authors:** Tietao Wang, Lin Zhang, Mijoon Lee, Wenbo Yan, Qian Liu, Chenchen Wang, Rhona Feltzer, Dusan Hesek, Shahriar Mobashery, Liang Zhang, Haihua Liang

**Affiliations:** 1 Key Laboratory of Resources Biology and Biotechnology in Western China, Ministry of Education, College of Life Sciences, Northwest University, Xi'an, Shaanxi, China; 2 Department of Pharmacology and Chemical Biology, School of Medicine, Shanghai Jiao Tong University, Shanghai, China; 3 Department of Chemistry and Biochemistry, University of Notre Dame, Notre Dame, Indiana, USA; 4 Department of Biochemistry, SUSTech Homeostatic Medicine Institute, School of Medicine, Southern University of Science and Technology, Shenzhen, China; 5 Key University Laboratory of Metabolism and Health of Guangdong, Southern University of Science and Technology, Shenzhen, China

**Keywords:** *Pseudomonas aeruginosa*, peptidoglycan hydrolase, antibacterial toxin, bacterial competition

## Abstract

*Pseudomonas aeruginosa* is an opportunistic pathogen that frequently resides in multispecies communities. During chronic infections, *P. aeruginosa* employs a diverse arsenal of antibacterial weapons to compete with other bacteria for resources and space. Using genetic and biochemical approaches, we identified a type VI secretion system–dependent antibacterial effector–immunity pair, PseM (*P. aeruginosa* secreted endolytic muramidase) and PA0990 in *P. aeruginosa*. Our findings demonstrate that PseM functions as an endolytic muramidase, targeting prey bacteria by hydrolytically cleaving cell-wall peptidoglycan, whereas its immunity partner PA0990 provides self-protection. The X-ray crystal structure of PseM reveals a homodimeric configuration, with its active site formed by segments from both monomers. Through structural analysis and macromolecular docking simulations, we further elucidate the substrate-binding residues critical for the activity of PseM. Importantly, we show that PseM contributes to *P. aeruginosa* growth among bacterial competition. Together, these results uncover a novel antibacterial mechanism mediated by PseM, highlighting the dynamic nature of interspecies and intraspecies competition within bacterial populations.

Bacteria commonly live in multispecies communities that allow them to survive, propagate, and compete in their microbiota (1). The intrabacterial and interbacterial cooperation and competition are important for stabilization and development of the microbial ecology (2). Bacteria cooperate with others for sharing extracellular public products or cell signaling molecules, which benefit the ecosystem; meanwhile, bacteria compete for nutrients and resources in environmental niches and limited living space (1, 3, 4). Bacteria use secretion systems to transport proteins, DNAs, or other macromolecules to the extracellular milieu or into host cells for their environmental and immunological adaptation *via* contact-dependent and contact-independent manners (5). In brief, the bacterial secretion systems play key roles in cooperation or competition for bacterial microecology in polymicrobial communities (6, 7).

Within microbial communities, interbacterial competition predominates as organisms vie for competitive advantages. During long-term evolution, bacteria have evolved diverse secretion systems, which play critical roles in their life cycles (8). The type VI secretion system (T6SS) exemplifies this adaptation, delivering effector proteins that target essential bacterial structures, including nucleic acids and cell walls, to mediate antagonistic interactions (5, 6). For instance, *Agrobacterium tumefaciens* and *Yersinia pseudotuberculosis* (*Yptb*) employ T6SS-translocated effectors Tde1 and Tce1, respectively, which suppress rival growth through nuclease activity (9, 10). *Serratia marcescens* utilizes its T6SS to deploy the effector Ssp6, which disrupts bacterial viability by inducing inner-membrane depolarization and enhancing outer-membrane permeability (11).

*Pseudomonas aeruginosa* is a ubiquitous opportunistic pathogen that affects a large number of patients with cystic fibrosis, lung diseases, burns, long-term intubated patients, and the immunocompromised individuals (12, 13). During chronic infections, *P*. *aeruginosa* typically resides in multispecies microbial communities where intense competition for resources and ecological niches occurs (14, 15). To gain a competitive advantage within these polymicrobial environments, *P. aeruginosa* has evolved an extensive arsenal of antibacterial weapons that target cohabiting microorganisms (16). For instance, two T6SS-dependent effectors, PldA and PldB, are translocated into eukaryotic cells, where they activate actin rearrangement and protrusion formation *via* the PI3K–Akt pathway, thereby facilitating host cell internalization (17). Most *P. aeruginosa* T6SS effectors function as antibacterial toxins, including Tses and Tles, which serve as molecular weapons in interbacterial and intrabacterial competition (18, 19). The Tae family amidase Tse1 specifically hydrolyzes peptidoglycan crosslinks, whereas the Tge family glycoside hydrolase Tse3 cleaves the glycosidic bond between *N*-acetylmuramic acid (MurNAc) and GlcNAc in the peptidoglycan backbone (20). Although numerous antibacterial effectors have been characterized, many remain to be identified.

In this study, we identified the product of the *P. aeruginosa* gene locus PA0989 as a T6SS-secreted endolytic muramidase (designated PseM for *P. aeruginosa* secreted endolytic muramidase), which specifically targets peptidoglycan in bacterial cell walls, leading to cell lysis. Crystallographic analysis reveals that PseM forms a homodimeric structure with an active site composed of residues from both monomers. Through macromolecular modeling, we have identified critical substrate-binding residues essential for the enzymatic activity of PseM. Importantly, we demonstrate that PseM significantly contributes to competitive fitness in bacterial interactions. These findings expand our understanding of the molecular diversity among bacterial-secreted antibacterial toxins.

## Results

### PseM is a T6SS-dependent secreted toxin in *P. aeruginosa*

**Figure 1:**
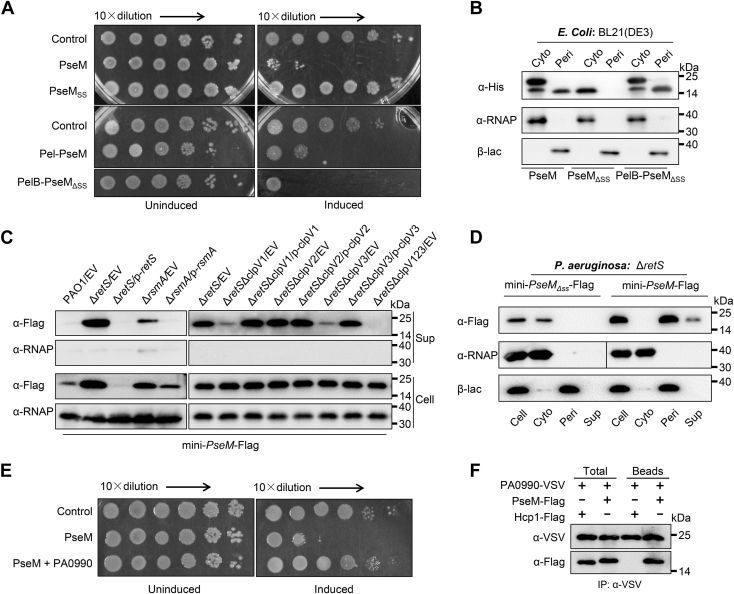
**PseM is a T6SS-dependent secreted toxin in *Pseudomonas aeruginosa*.***A,* growth of *Escherichia coli* BL21 (DE3) pLysS strains expressing empty vector (control), full-length PseM, PseM lacking its signal peptide (PseM_ΔSS_), or PelB–PseM_ΔSS_ on LB agar with or without 1.0 mM IPTG at 37 °C for 24 h. *B,* Subcellular localization of PseM variants. Cytoplasmic (Cyto) and periplasmic (Peri) fractions of *E. coli* expressing PseM, PseM_ΔSS_, or PelB–PseM_ΔSS_ were analyzed by Western blot using α-His antibody. RNA polymerase (α-RNAP) and β-lactamase (β-lac) served as compartment-specific controls. *C,* RetS-mediated T6SS regulation of *pseM*. Western blot of PseM-FLAG in culture supernatant (Sup) and cell pellet (Cell) fractions from *P. aeruginosa* strains. α-RNAP antibody was used as a loading control. *D,* role of the N-terminal signal peptide in PseM secretion. Cell, Cyto, Peri, and Sup fractions of *P. aeruginosa* Δ*retS* strains expressing PseM-FLAG or PseM_ΔSS_-FLAG were probed with α-FLAG antibody. Compartment markers: α-RNAP (Cyto) and β-lac (Peri). *E* and *F,* PA0990 is an autoimmunity protein for PseM. *E,* growth of *E. coli* strain BL21 (DE3) pLysS harboring an empty vector (Control), expressing PseM, or PseM and PA0990 (PseM+PA0990) on LB agar plates with or without IPTG at 37 °C. *F,* co-IP assays showing PA0990 binds to PseM. Overnight cultures of *P. aeruginosa* containing pMMB67HE-*PA0990*-VSV with either mini-CTX-*pseM*-FLAG or mini-CTX-*hcp1*-FLAG were lysed. Cell-lysis supernatants were incubated with anti-VSV beads, and then the bead-coated proteins were detected by Western blot with α-FLAG or α-VSV antibody. Hcp1-FLAG was a negative control. *A–F,* representative data from three biological replicates. EV, empty vector; PseM, *P. aeruginosa* secreted endolytic muramidase; VSV, vesicular stomatitis virus.

Bioinformatic analysis revealed that PseM encodes a hypothetical protein of unknown function containing an N-terminal signal peptide (Fig. S1*A*) (21). Since some Sec-dependent secretory proteins are associated with bacterial toxin (22, 23) we generated a cytoplasmic (Cyto) expression plasmid (pET28a-*pseM*) and transformed it into *Escherichia coli* BL21(DE3) pLysS. Heterologous expression under an IPTG-inducible promoter demonstrated that PseM inhibits *E. coli* growth (Figs. 1*A* and S1*B*). To investigate whether the N-terminal signal peptide contributes to this toxicity, we engineered two constructs: PseM_ΔSS_ (signal peptide–deleted PseM) and PelB–PseM_ΔSS_ (PseM_ΔSS_ cloned into pelB signal peptide–containing pET22b). Cyto production of PseM_ΔSS_ failed to inhibit bacterial growth (Figs. 1*A* and S1*C*), whereas PelB–PseM_ΔSS_ induction exhibited antibacterial activity equivalent to full-length PseM overexpression (Figs. 1*A* and S1*C*). These results demonstrate that the signal peptide is essential for PseM-mediated toxicity in *E. coli*. To determine PseM localization, we performed subcellular fractionation followed by Western blot analysis. Both PseM and PelB–PseM_ΔSS_ localized to the periplasmic (Peri) compartment, whereas PseM_ΔSS_ remained Cyto (Fig. 1*B*).

RetS is a hybrid sensor kinase that regulates bacterial secretion systems and virulence factors in *P. aeruginosa* infection, including the type III secretion system, T6SS, motility, biofilm formation, and exopolysaccharide production (24). Importantly, we observed that both expression and secretion of PseM were negatively regulated by RetS (Fig. 1*C*). RsmA, a translational repressor that inhibits target genes by binding to mRNA, is systematically regulated through the RetS–Rsm signaling cascade (25). Consistent with this regulatory network, RsmA was found to repress both expression and secretion of PseM (Fig. 1*C*). To further investigate this regulation, we analyzed the upstream sequence of *pseM* using Mfold (26), which revealed a predicted RsmA-binding motif upstream of *pseM* (Fig. S1*D*). These findings demonstrate that PseM expression and secretion are directly regulated by the RetS–Rsm system.

Since T6SS is strictly controlled by the RetS–Rsm signaling cascade (27), we hypothesized that the PseM secretion may be dependent on T6SS. To verify this hypothesis, we generated mutant strains lacking *retS* combined with deletions of *clpV1*(ΔretSΔclpV1), *clpV2* (Δ*retS*Δ*clpV2*), *clpV3* (Δ*retS*Δ*clpV3*), or all three *clpV* genes (Δ*retS*Δ*clpV123*), as ClpV proteins form essential structural components of the T6SS apparatus. Intriguingly, Western blot analysis revealed reduced PseM secretion in Δ*retS*Δ*clpV1* and Δ*retS*Δ*clpV3* strains compared with the Δ*retS* single mutant, with complete abolition of secretion in the Δ*retS*Δ*clpV123* triple mutant (Fig. 1*C*). Given the 'Peri localization of PseM in *E. coli*, we investigated its subcellular targeting and secretion in *P. aeruginosa* by constructing FLAG-tagged PseM and PseM_ΔSS_ expression plasmids (mini-CTX-*pseM*-FLAG and mini-CTX-*pseM*_*ΔSS*_-FLAG) and integrating them into the Δ*retS* mutant. Subcellular fractionation confirmed Peri localization and extracellular secretion of full-length PseM but not the signal peptide–deficient PseM_ΔSS_ (Fig. 1*D*). Collectively, these results establish PseM as a T6SS-dependent secreted bacterial toxin in *P. aeruginosa*.

The genes for T6SS bacterial toxin effectors are often contiguous with those of the antitoxin proteins for self-protection (20). Through genomic analysis of the region upstream of *pseM* (PA0989) gene in *P. aeruginosa* PAO1 genome, a putative immunity gene, *PA0990*, was found. To explore whether PA0990 encodes an immunity protein for PseM, we evaluated the effect of the production in *E. coli* BL21(DE3) pLysS of both PseM and PA0990 proteins using the dual expression plasmid pET-Duet-1. Under IPTG induction, coproduction of PA0990 and PseM abrogated the growth inhibitory activity of PseM (Fig. 1*E*). Autoimmunity proteins abrogate the activity of their corresponding toxin proteins by direct protein–protein interaction (20). An *in vivo* coimmunoprecipitation (co-IP) assay using vesicular stomatitis virus (VSV) glycoprotein tag was carried out to document the interaction between PseM and PA0990. Bacterial cultures of *P. aeruginosa* coproducing PA0990-VSV, PA0990 protein fused with a VSV affinity tag, with either PseM-FLAG or Hcp1-FLAG (a negative control) were lysed, and the supernatants (Sups) were further purified by anti-VSV beads. The presence of proteins was detected by Western blot against α-FLAG or α-VSV antibody. Data showed that PseM was able to bind to PA0990 but not to Hcp1 (Fig. 1*F*). These data demonstrated that PA0990 is an autoimmunity protein of PseM by direct interaction.

### PseM functions as an endolytic muramidase

**Figure 2:**
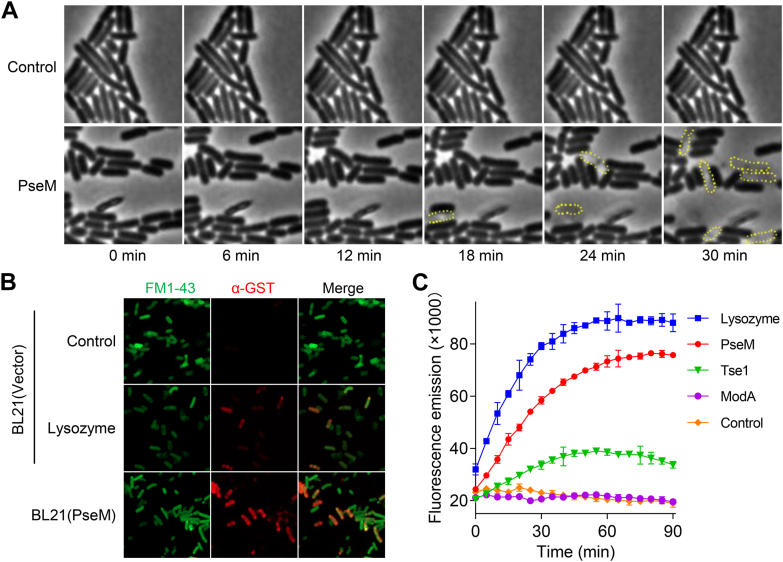
**PseM induces cell lysis in *Escherichia coli*.***A,* a time-lapse microscopy demonstrating PseM-mediated *E. coli* lysis. *E. coli* cells were immobilized on LB–LS agarose pads containing 1 mM IPTG and imaged at 37 °C for 30 min using a Nikon Ti2-E inverted microscope. Lysed cells (*yellow dashed circles*) are shown in representative frames from Movies S1 and S2. *B,* outer-membrane integrity analysis. *E. coli* BL21 (DE3) strains (producing glutathione-*S*-transferase [GST]) harboring empty vector pET28a (BL21(Vector)) or pET28a-*pseM* (BL21(PseM)) were grown to an absorbance of 0.6 at 600 nm in LB supplemented with 0.1 mM IPTG. Cells were fixed, mock treated, or lysozyme treated, then incubated with anti-GST primary antibodies and Alexa Fluor 647-conjugated secondary antibodies. Membranes were counterstained with FM1-43 dye and visualized by microscopy (full images in Fig. S2). The data in *A* and *B* are representative of three independent replicates. *C,* enzymatic activity quantification using the EnzChek Lysozyme Assay Kit. PseM lytic activity was compared with positive controls (egg lysozyme, T6SS secreted effector Tse1) and a negative control (molybdate-binding protein ModA). Data in *A* and *B* are representative of three biological replicates; (*C*) shows mean ± SD (*n* = 3). GST, glutathione-*S*-transferase; PseM, *P. aeruginosa* secreted endolytic muramidase; T6SS, type VI secretion system.

As stated previously, the toxic activity of PseM is dependent on its Peri sequestration, and as we will show, its activity is manifested on the cell-wall peptidoglycan. To visualize the bacterial cell-wall disintegration, time-lapse analysis for bacterial cell morphology with microscopy was carried out. As shown in the video, *E. coli* production of PseM leads to cell lysis or burst (Fig. 2*A* and Movies S1-S2). Cell-wall degradation is often followed by outer-membrane permeability transition, so we sought to test whether PseM is associated with this function. *E. coli* coproducing PseM and glutathione-*S*-transferase (GST) were incubated with GST primary antibody and Alexa-Fluor-647–conjugated secondary antibody, and then followed with cell-membrane dye FM1-43. Microscopy showed that, similar to lysozyme-treated cells, the corresponding antibodies could permeate through the envelope of PseM-producing strains (Figs. 2*B* and S2). These results indicated that production of PseM increased the cell-membrane permeability, preceded by lysis.

**Figure 3:**
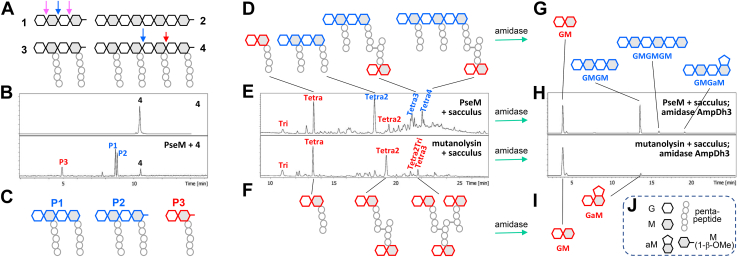
**PseM is an endolytic muramidase with a minor exolytic activity.***A,* four synthetic peptidoglycans used in this study (**1**–**4**). *B* and *C,* reaction of peptidoglycan **4** with PseM and the respective products. *D*–*F,* reactions of PseM (*upper panel E*) or mutanolysin (*lower* in *E*) with the *Escherichia coli* sacculus and reaction products. *G*–*I,* reactions of amidase AmpDh3 of PseM-predigested sacculus (*top panel*, *H*) or mutanolysin-predigested sacculus (*bottom panel*, *H*) and corresponding reaction products. *J, cartoon annotations* used in this figure. G = GlcNAc, M = *N*-acetyl muramic acid, aM = *N*-acetyl 1,6-anhydromuramic acid, and pentapeptide = l-Ala^1^-d-γ-Glu^2^-*m*-DAP^3^-d-Ala^4^-d-Ala^5^. The full peptide stem in *E. coli* is a pentapeptide, and this can be shortened to Tetra, Tri, and Di from the C terminus. *Pink and blue arrows* in compound **1** in *panel A* indicate the cleavage sites for glucosaminidase and muramidase or lytic transglycosylases, respectively; *blue and red arrows* in compound **4** in *A* indicate the major and minor cleavage sites of PseM. N-acetylmuramic acid moiety at the reducing end was reduced by sodium hydride after enzymatic reactions (*D*–*I*). Structures shown in *red* were found in both PseM and mutanolysin reactions, and structures shown in *blue* were uniquely found in the reaction of PseM with the sacculus and are an indication of endolytic reactions (*D*, *E*, and *G*). *B, E*, and *H,* data are representative of two independent replicates. PseM, *P. aeruginosa* secreted endolytic muramidase.

We next explored if PseM could degrade cell-wall peptidoglycan *in vitro*. As a first-pass experiment, we assayed for peptidoglycan-degrading activity of PseM by the fluorescent EnzChek Lysozyme Assay Kit. The data showed that PseM indeed exhibited peptidoglycan-degrading activity (Fig. 2*C*). To elucidate the exact nature of the enzymatic activity, four synthetic peptidoglycans (compounds **1**–**4**) were used (Figs. 3*A* and S3*A*). Their syntheses were based on the methodology developed by the Mobashery laboratory earlier (28, 29, 30). Two substrates contain four linear sugars (GMGM, where G = GlcNAc, M = MurNAc) without and with pentapeptide stems (compounds **1** and **3**, respectively; pentapeptide, l-Ala-γ-d-Glu-*m*-Dap-d-Ala-d-Ala). The remaining two substrates contain eight linear sugars 2× (GMGM) without and with pentapeptide (compounds **2** and **4**, respectively). If the enzyme were to be a glucosaminidase, it would cleave the bonds indicated with *pink arrows* in compound **1** (Fig. 3*A*). If it were a muramidase or a lytic transglycosylase, it would cleave the bond indicated with the *blue arrows* (Fig. 3*A*). We set up reactions of PseM with the four synthetic peptidoglycans, and the reaction mixture was analyzed by ultra-performance liquid chromatography–mass spectrometry (UPLC–MS). Among the four synthetic peptidoglycans, only compound **4** was turned over by PseM, and two major products (P1 and P2) were formed (Figs. 3, *B* and *C* and S3*B*). Based on the UPLC–MS result, product P2 was compound **3**; product P1 has four sugars with two pentapeptide stems. The formation of P1 indicates that PseM is a muramidase, not a lytic transglycosylase reaction (31). The disaccharide product P3 was also found as a minor component (Figs. 3, *B* and *C* and S3*B*), formed by cleavage of the bond indicated with a *red arrow* on compound **4** in Figure 3*A*. Overall, PseM hydrolyzes substrates with peptide stems, and it prefers the ones with a longer sugar backbone, indicating that it is an endolytic muramidase with a minor exolytic activity, as shown with the synthetic peptidoglycan **4**.

To complement the experiment with synthetic peptidoglycans, reactions of PseM with the purified *E. coli* sacculus were set up. Reactions of mutanolysin, a known exolytic muramidase, were set up as a control (Figs. 3, *D*–*F* and S3*C*). Products of mutanolysin reactions mainly contain disaccharide units, GlcNAcMurNAc or GM, with non–crosslinked stem peptides or crosslinked peptides given in *red color* (Figs. 3, *E* and *F* and S3*C*). The reaction of PseM with the sacculus produced similar products as those of mutanolysin (Figs. 3, *D* and *F* and S3*C*). But PseM also produced unique products colored *blue*, and those products contained linear tetrasaccharide or hexasaccharide backbones with non–crosslinked or crosslinked peptide stems (Figs. 3, *D* and *F* and S3*C*). However, the peptide stems of the products make the profile more complex and challenging for analysis. Therefore, the reaction products of PseM were further subjected to transformation by the peptidoglycan amidase AmpDh3, which cleaves the bond between lactate and l-Ala of the stem peptide (32). Indeed, elimination of the stem peptides from the saccharide components simplified the profile significantly, and the result clearly showed the formation of disaccharides and tetrasaccharides (GM and GMGM) as major products (Figs. 3, *G*–*I*, S3*D* and S4). Prominent exolytic activity of mutanolysin produced only disaccharides (GM and GaM) under the same conditions (Figs. 3, *H* and *I*, S3*D* and S4). Overall, reactions of PseM with sacculus indicate that PseM is an endolytic muramidase, with some exolytic activity.

### Crystal structure of *P. aeruginosa* PseM

**Figure 4:**
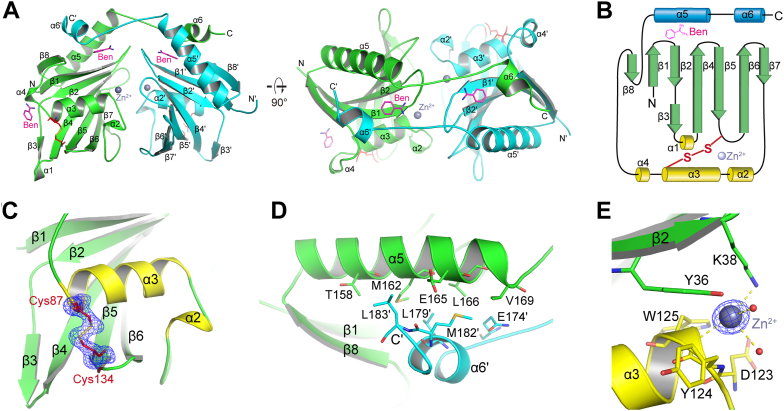
**Crystal structure of PseM.***A,* the X-ray crystal structure of the homodimeric PseM from *Pseudomonas aeruginosa*. The monomers of PseM within the homodimer were colored in *green* (monomer A) and *cyan* (monomer B). The benzamidine (Ben) molecules and the disulfide bonds were shown in *capped sticks* and colored in *magenta* and *red*, respectively. The zinc ions were shown as *light blue spheres*. The secondary structures of PseM are depicted. The prime indicates that the residue is from monomer B. *B,* the topology diagram of PseM. *C,* the fo–fc omit electron density map contoured at 3.0 σ around the disulfide bond (Cys87–Cys134). *D,* the dimerization of two PseM monomers. The residues involved in the dimerization were shown in *sticks* and labeled. *E,* the fo–fc omit electron density map contoured at 3.0 σ around the zinc ion. The residues that form interactions with the metal ion were shown in *capped sticks* and labeled. PseM, *P. aeruginosa* secreted endolytic muramidase.

We determined the crystal structure of PseM. Per an earlier comment, sequence analysis suggested that the N terminus has a signal peptide (residues 1–21, cleavage site between residues 26 and 27) (Fig. S1*A*). Accordingly, the truncated gene for PseM (residues from 28 to 186) was cloned, and the corresponding protein was purified to homogeneity (Fig. S5*A*) and concentrated to 27 mg/ml for crystallization. After approximately 2 weeks of incubation, tube-shaped crystals were observed in the reservoir solution containing 0.1 M Mes monohydrate, pH 7.0, 2.0 M NaCl, and 1% benzamidine (Ben)–HCl. The crystals were subsequently soaked in protectant containing mother liquor supplemented with 30% glycerol (v/v) and flash frozen in liquid nitrogen for X-ray diffraction. The structure of PseM was subsequently solved by single-wavelength anomalous diffraction, and a pair set of 2.0 Å diffraction data for the selenomethionine substitution or native PseM protein were collected with the space group P4_3_2_1_2 (Table S1). The structure determination revealed a homodimer in the asymmetric unit (Fig. 4*A*). The two monomers dimerized in a head-to-tail arrangement by mostly hydrophobic interactions with the C-terminal α-helices.

Analysis by the DALI server (http://ekhidna2.biocenter.helsinki.fi/dali/) revealed a unique structure for PseM; no reported structural homolog was found (33). The PseM monomer consisted of a β-sheet core comprised of eight antiparallel β-strands with topology β8 (residues 145–150)-β1 (30–38)-β2 (45–50)/β3 (55–57)-β4 (61–70)-β5 (78–86)-β6 (91–100)-β7 (107–109), and stacked by α-helices on both sides (Fig. 4*B*). An α-helix bundle consisting of N-terminal α1 (58–60), α2 (119–121), α3 (123–134), and α4 (136–138) helices stabilizes the β-sheet core on one side with a disulfide bond between Cys87 and Cys134 located on α3 and β5, respectively (Fig. 4*C*). In contrast, on the reverse side of the β-sheet core, C-terminal α5 (152–169) and α6' (176–181) from the other monomer within the homodimer interact with each other *via* interactions from α5 (Thr158, Met162, Glu165, Leu166, and Val169) and from α6' (Glu174′, Leu179′, Met182′, and Leu183′). (The prime indicates that the residue is from monomer B) (Figs. 4, *A* and *D*).

A zinc ion is coordinated by the side chain of Lys38 on β2, the backbone nitrogen of Tyr125 on α3, and two water molecules. In addition, hydrophobic interactions occur between the zinc ion and the side chain of Tyr36 on β2, as well as the side chains of Asp123, Tyr124, and Trp125 on α3, positioned between the N terminus of α3 and the C terminus of β2 (Fig. 4*E*). As expected, *in vitro* analysis shows that the zinc ion is important for the peptidoglycan-hydrolytic activities of PseM (Fig. S5*B*). Moreover, the dimer interface forms a crevice that sequesters a Ben molecule from the crystallization solution through hydrophilic interactions. These interactions involve the side chain of Asn44 on the α4-helix and the backbone oxygen atom of Gly39 (located on a loop between β1 and β2) from one monomer as well as the backbone nitrogen atom of Gln174′ from the adjacent monomer (Fig. S5*C*). The Ben is further stabilized by hydrophobic interactions with the side chains of Gly37, Val46, Met162, Met176′, and Leu179'.

### Characterization of the catalytic mechanism of PseM

**Figure 5:**
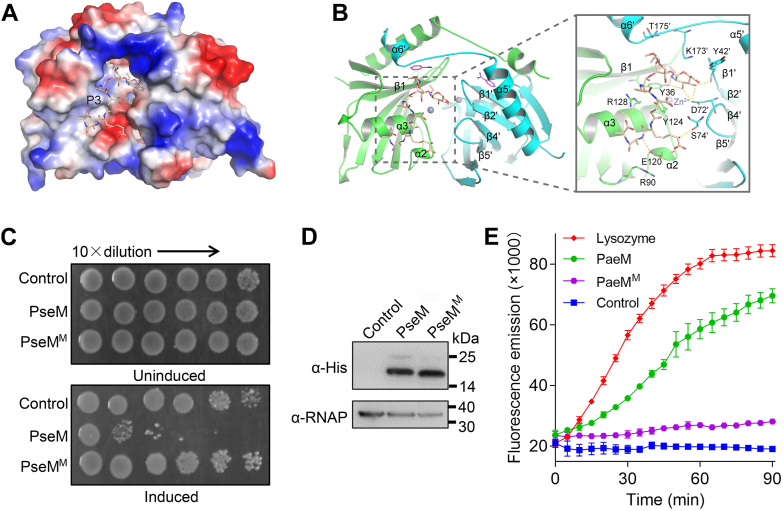
**Characterization of PseM binding to its substrate.***A* and *B,* molecular docking of substrate P3 to the PseM homodimer. The substrate is depicted as *wheat-colored sticks*, and the residues involved in the binding of the substrate are shown in *sticks* and labeled. *Yellow dashed lines* indicate H-bonds. *C,* functional assessment of quadruple mutant PseM (PseM^M^, PseM^Y36A/R90A/E120A/R128A^). Growth of *Escherichia coli* strain BL21 (DE3) pLysS expressing empty vector (Control), wildtype PseM (PseM), or mutant PseM (PseM^M^) on LB agar plates with or without 1.0 mM IPTG at 37 °C. *D,* comparable expression levels of PseM and PseM^M^ in *E. coli*, confirmed by immunoblot analysis. *E,* the peptidoglycan hydrolytic activity of PseM and PseM^M^ was detected by the fluorescence method using the EnzChek Lysozyme Assay Kit. Error bars represent the mean ± SD of three biological replicates. *C* and *D,* data are representative of three independent replicates. PseM, *P. aeruginosa* secreted endolytic muramidase.

To investigate the catalytic mechanism, we determined the activity of PseM after preparing amino-acid variants based on the information from the crystal structure, which included the corresponding amino acids: Cys87, Cys134, Tyr36, Glu120, Lys173, Arg90, Tyr124, Arg128, Lys38, Asn43, and Asp123 with single-point mutations; Cys87/Cys134, Tyr36/Glu120, Tyr36/Arg128, and Tyr36/Arg90 with double-point mutations; and Arg90/Glu120/Arg128 and Tyr36/Tyr124/Lys173 with three-point mutations; however, all these PseM variants still displayed antibacterial toxicity (Fig. S6). In the light of the crystallographic lack of success for the complex with peptidoglycan samples, the product P3 (GM-pentapeptide) was docked to the PseM dimer structure based on macromolecular docking (34). As shown in Figures 5, *A* and *B*, the P3 ligand binds to the crevice around β1 and α3 near the dimer interface and the zinc ion–binding site of PseM and is predominantly stabilized by electrostatic interactions with surrounding residues (Figs. 5, *A* and *B*). The NAG is stabilized by hydrogen bonds formed with the side chains of Asp72′ from the loop between β4′ and β5′, the backbone oxygen atoms of Tyr42′ from the loop between β1′ and β2′, and the side chain of Tyr36 from β1. The pentapeptide of P3 forms electrostatic interactions with the side chains of Glu120 from α2, Arg128 from α3, and the backbone oxygen atoms of Ser74′ from the loop between β4′ and β5'. In addition, the ligand benefits from hydrophobic interactions with the side chains of Tyr36, Tyr124, and Arg128 from α3, Tyr42′, Asp72′, and Lys173′ from the loop between α5′ and α6′.

To further validate the key residues involved in P3 binding, we engineered a series of site-specific PseM mutations guided by macromolecular docking, including single, double, triple, and quadruple mutants, and assessed their bacterial toxicity. As shown in Figures 5 and S6, only simultaneous mutagenesis of all four P3-binding residues (Tyr36/Arg90/Glu120/Arg128) abolished PseM's toxic activity *in vivo*, whereas PseM^M^ maintained protein levels in *E. coli* comparable to wildtype PseM (Figs. 5, *C* and *D*, and S6). Consistently, the variant protein with four key residues loses the peptidoglycan hydrolytic activities (Fig. 5*E*).

### PseM contributes to interspecies competition

**Figure 6:**
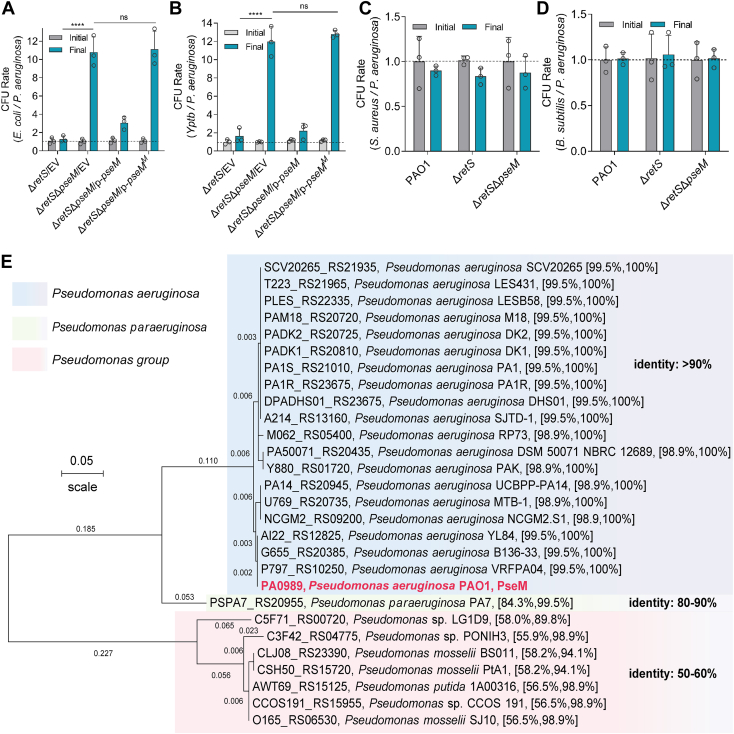
**PseM is essential for *Pseudomonas aeruginosa* in interbacterial competition.** Interspecies competition assays between the indicated *P. aeruginosa* and competitor species: *Escherichia coli* (*A*), *Yersinia pseudotuberculosis* (*Yptb*) (*B*), *Staphylococcus aureus* (*C*), and *Bacillus subtilis* (*D*). Competitor strains were cocultured with *P. aeruginosa* strains for 24 h. Colony-forming unit (CFU) ratios (competitor *versus P. aeruginosa*) were calculated from initial and final timepoints. Data represent mean ± SD of triplicate biological replicates. Statistical significance was calculated using the one-way ANOVA Dunnett's multiple comparison test, ∗∗∗∗*p* < 0.0001; ns, not significant. *E,* the phylogenetic relationship of PseM. Phylogenetic analysis of PseM homologs. The evolutionary tree includes PseM (locus tag PA0989) and homologs with >90% sequence identity. PA0989 homologs with 50% to 60% identity were identified in other *Pseudomonas* species. Evolutionary distances are indicated at branch nodes. EV, empty vector; PseM, *P. aeruginosa* secreted endolytic muramidase.

Bacterial toxins are often involved in bacterial population competitions. To assess whether PseM contributes to interbacterial competition *in vitro*, a coincubation assay of the *P. aeruginosa* strain and *E. coli*, *Yptb*, *Staphylococcus aureu*s, or *Bacillus subtilis* as a competitor was performed. Data showed a significant increase in the number of cell rate (competitor/*P. aeruginosa*) of the Δ*retS*Δ*pseM* mutant strain compared with the single Δ*retS* mutant (Figs. 6, *A* and *B*). In addition, the competitive advantage of *P. aeruginosa* Δ*pseM* was restored to the wildtype levels by introducing the p-*pseM* plasmid, but not the p-*pseM*^*M*^ plasmid, which resulted in a loss of peptidoglycan hydrolase activity (Figs. 6, *A* and *B*). However, there are no significant differences when coincubation of Δ*retS*Δ*pseM* with two Gram-positive bacteria, *S. aureus* and *B. subtilis*, compared with Δ*retS* strain (Figs. 6, *C* and *D*). Together, these data reveal the effect of the bacterial toxin PseM on the competitor strain, thus degrading cell-wall peptidoglycan and providing a growth advantage for *P. aeruginosa* in competition with other bacteria, especially to Gram-negative bacteria.

### The PseM homologs are ubiquitous in *Pseudomonas*

To investigate the evolutionary relationships of PseM, we performed phylogenetic analysis using the neighbor-joining method in MEGA with identified PseM homologs. A BLASTp search revealed 28 PseM-like candidates (Fig. 6*E*), which were subsequently included in the phylogenetic reconstruction. The analysis demonstrated that PseM homologs are widely distributed across *Pseudomonas* strains. Notably, PSPA7_RS20955, a PseM homolog from strain PA7, formed an outgroup relative to other *P. aeruginosa* strains. As expected, sequence identity among *P. aeruginosa* homologs was significantly higher than that observed in homologs from other species.

## Discussion

Bacterial secretion systems deliver toxic effector molecules to neighboring organisms within shared ecological niches to confer competitive advantages (6). Here, we report the discovery of a novel antibacterial toxin, PseM, secreted by T6SS of *P. aeruginosa*. Our findings demonstrate that PseM functions as an endolytic muramidase that specifically targets peptidoglycan in the cell walls of competitor bacteria. This enzymatic activity enables *P. aeruginosa* to eliminate rival microbial populations, thereby providing a distinct fitness advantage through niche domination.

*P. aeruginosa* encoded three peptidoglycan amidases, AmpD, AmpDh2, and AmpDh3 (32, 35). AmpD is a cytosolic *N-acetyl-anhydromuramyl*-l-alanine amidase, which hydrolyzes 1,6-anhydromuramylpeptides and acts in antibiotic resistance and cell-wall recycling (35). AmpDh2 and AmpDh3 are Peri enzymes, which exhibited marginal activities with the 1,6-anhydromurmyl and function in turnover of cell wall (32). Our previous study revealed that AmpDh3 hydrolyzes the cell-wall peptidoglycan of the prey bacterium (36). Tse1 and Tse3 are two T6SS-secreted antibacterial effectors of *P. aeruginosa,* in which Tse1 is a peptidoglycan amidase (dl-endopeptidases) (20). Where Tse3 is a glycoside hydrolase, which cleaves the glycan backbone between MurNAc and GlcNAc of peptidoglycan (20). Here, we revealed a novel *P. aeruginosa*-secreted antibacterial toxic PseM, which exhibited an endolytic muramidase and a minor exolytic activity. The enzyme prefers the presence of the stem peptide in the substrate, which indicates that it targets peptidoglycan along the length of the bacterial cell wall, as opposed to the polar caps (37).

Bacteria have developed multiple mechanisms to communicate and compete with one another in complex environments. Many bacteria use the contact-dependent growth inhibition systems to gain growth advantage in bacterial competition *via* secreting the toxic protein, CdiA (38). Another well-studied example with an important function in bacterial competition is T6SS. *Yptb* exploiting T6SS-dependent effector TepC provides a competitive advantage for itself in the microecology *via* its iron-binding ability and DNase activity (39). In addition, T6SS also plays a critical role in the intestinal microbiota competition. *Shigella sonnei* encodes a T6SS that provides a competitive advantage in the gut. Moreover, *S. sonnei* can persist as well as outcompete *E. coli* and *S. flexneri* in mice in a T6SS-dependent manner (40). Here, we found that the PseM of *P. aeruginosa* functions as a toxin among interbacterial growth competition with *E. coli* and *Yptb* (Figs. 6, *A* and *B*), indicating that the endolytic muramidase PseM may serve as a potential tool for regulating the microbiota composition in multibacterial infections or inflammatory disease.

The protein PseM contains an N-terminal signal peptide and is generally secreted to the cell periplasm space *via* the Sec system (41). Intriguingly, experimental results found that PseM secretion depends on both the T6SS and its signal peptide—a unique feature among T6SS effectors, as they typically lack signal peptides. This suggests that PseM employs a distinct dual mechanism for secretion through the T6SS pathway. The *Vibrio parahaemolyticus* exotoxin thermostable direct hemolysin is secreted by both the type II secretion system and type III secretion system utilizing dual signal peptides (42), which shows a multifaceted secretion strategy. In *P. aeruginosa*, PseM is secreted by the T6SS in a subcellular localization–dependent manner. This suggests a novel T6SS secretion pattern in bacteria, in which PseM cargo may be loaded onto the T6SS apparatus carrier in the periplasm during system assembly prior to T6SS firing (43, 44). This implies an unprecedented T6SS secretion mechanism, where a signal peptide–bearing effector is co-opted from the periplasm for extracellular delivery. In addition, T6SS-secreted effector proteins are generally accompanied by cognate immunity proteins to prevent self-toxicity. In this study, genomic analysis revealed that *PA0990*, located upstream of the *pseM* (*PA0989*) gene, serves as the immunity protein for PseM through direct binding. Notably, PA0990 contains a predicted Nudix_hydrolase domain, suggesting it functions as an ADP-ribosyl hydrolase. This implies that PseM may exert its toxicity *via* ADP-ribosylation, whereas PA0990 counteracts this activity through its hydrolase function. These findings establish PseM as a novel Peri T6SS–secreted toxin, this proposed pathway distinct from canonical T6SS effector loading, whose secretion and detoxification mechanism warrants further investigation.

To investigate the catalytical mechanism of PseM, the high-resolution (2.0 Å) crystal structure of PseM was subsequently determined (Fig. 4). PseM exhibits a homodimer oligomerization, in which the monomer displays a unique two α-helix bundles stacked and an antiparallel β-sheet core that has never been observed through published enzymes so far, and a zinc ion binds between the N terminus of α3 and the C terminus of β2 of PseM to stabilize the folding of the structure. Further docking results suggested the P3 product binds to the crevice around β1 and α3 near the dimer interface and the zinc ion–binding site of PseM, which is predominantly stabilized *via* hydrophilic interactions with residues around. Based on the docking results, serials of site-point mutant PseM analysis show only four site-point variants, PseM^M^, exhibited nontoxic catalytic, this implied that the substrate binding activity of PseM is dependent on multisite (Fig. 5).

Collectively, we characterize a new type of antibacterial effector, PseM, secreted by *P. aeruginosa* PAO1, which mediates bacterial competition through targeted peptidoglycan degradation. These findings reveal an additional ecological adaptation strategy employed by *P. aeruginosa* to dominate challenging environments, establishing a previously unrecognized antimicrobial mechanism in microbial warfare.

## Experimental procedures

### Bacterial strains and growth conditions

*P. aeruginosa*, *E. coli*, and *Yptb* strains and plasmids used in the study are listed in Table S2. Bacterial strains were grown in LB (1% tryptone, 0.5% yeast extract, and 0.5% NaCl) at 37 °C with appropriate agar or antibiotics. Antibiotics were used at the following concentrations: for *P. aeruginosa*, carbenicillin at 300 μg/ml and tetracycline at 100 μg/ml; for *E. coli*, carbenicillin at 100 μg/ml, kanamycin at 50 μg/ml, tetracycline at 10 μg/ml, and gentamicin at 25 μg/ml; and for *Yptb*, gentamicin at 25 μg/ml.

### Construction of plasmids and *P. aeruginosa* deletion mutants

Primers used in our study are listed in Table S3 and synthesized by Beijing Tsingke Biotech Co, Ltd. For bacterial toxic assay, plasmids pET28a-*pseM*, pET28a-*pseM*_*ΔSS*_, pET22b-*pseM*, and pET22b-*pseM*_*ΔSS*_ were constructed by PCR amplification using the indicated primer pairs, and the resulting gene fragments were digested and inserted into pET28a and pET22b plasmids, respectively. Plasmids pAK-*pseM*, pAK-*pseM*^*M*^, pET-Duet-*pseM*, and pET-Duet-*pseM-PA0990* were constructed in a similar manner. Plasmid mini-CTX-*pseM*-FLAG was constructed for Western blot assay. Briefly, primer CTX-*pseM*-S/A was used to amplify the *pseM* gene and its promoter region from the genomic DNA. The PCR product was digested and inserted into mini-CTX to generate mini-CTX-*pseM*-FLAG. Plasmid mini-CTX-*pseM*_*ΔSS*_-FLAG was constructed in a similar manner.

Site-directed mutagenesis of pET28a-*pseM*^*Y36A*^ was carried out by overlap PCR as described previously (45). Briefly, DNA for site-directed mutagenesis of *pseM* was amplified by two rounds of PCR. Primer pairs pET-*pseM*-S/pET-*pseM*^*Y36A*^-A and pET-*pseM*^*Y36A*^-S/pET-*pseM*-A were used to amplify segments 1 and 2, respectively. The next round of PCR was carried out using pET-*pseM*-S/A as a primer pair and segments 1 and 2 as templates to obtain the *pseM*^*Y36A*^ fragment. The resultant fragment was digested and cloned into similarly digested pET28a to produce pET-*pseM*^*Y36A*^. The other site-directed mutagenesis plasmids were constructed in a similar manner.

Markerless gene in-frame deletion of *P. aeruginosa* mutant strains was performed as described previously (36) to construct the *pseM* knock-out plasmid pEX18ap-*pseM*; the upstream fragment and downstream fragment of *pseM* were amplified using the primer pairs pEX-*pseM*-Up-S/A and pEX-*pseM*-Down-S/A, respectively. The resulting PCR products were digested with the indicated restriction enzymes and then inserted into pEX18ap to produce pEX18ap-*pseM*. The plasmid pEX18ap-*pseM* was electroporated into *P. aeruginosa* parental and selected with carbenicillin. The resultant single-crossover recombination strains were further cultured, and colonies showed both sucrose resistance and carbenicillin susceptibility, indicating double-crossover for gene in-frame deletion. The mutant strains were verified by PCR and DNA sequencing.

### Bacterial growth assays

*E. coli* BL21(DE3) pLysS harboring the pET series plasmids were grown to the stationary phase in liquid LB medium at 37 °C. The cell density of bacterial cultures was determined and serially diluted by 10-fold in LB medium. Then 5 μl of bacterial dilution was spotted on an LB-LS agar plate with the indicated concentration of IPTG. The growth results were assessed after 16 h at 37 °C. For growth curves, the bacteria were subcultured to a starting absorbance of 0.1 at 600 nm in LB. Bacteria were further cultured with the indicated concentration of IPTG, and the absorbance at 600 nm measurements were made for 10 h.

### Subcellular fractionation assay

Subcellular fractionation assays were performed according to the described methods (20, 46). The indicated *E. coli* or *P. aeruginosa* were grown to an absorbance of 1.5 at 600 nm in the given culture condition, at which point a 10-ml aliquot was diluted to 500 μl of 20 mM PBS, pH 7.3, 20% sucrose, and 2.5 mM EDTA. After incubation at room temperature for 20 min, the same volume of ice-cold distilled–deionized water was added and gently shaken for another 5 min. The samples were centrifuged (7000*g* for 20 min at 4 °C). Each 1 ml of the Sup was mixed with 180 μl of 100% trichloroacetic acid and incubated on ice for 1 h. The centrifuged protein pellet was washed with ice-cold acetone twice and resuspended in 50 μl of SDS-loading buffer. This sample was defined as Peri. The pellet was resuspended in 50 μl of SDS-loading buffer; this sample was defined as Cyto.

### Secretion and Western blot analysis

PseM-secretion assays were performed according to the described methods (27). Briefly, strains were inoculated in 5 ml of LB medium and incubated until an absorbance reached 0.9 at 600 nm at 37 °C. A total of 2 ml of the culture was centrifuged, and the Sup was filtered through a 0.22 μm PES filter (Millipore). For each 1 ml of the Sup, 180 μl of 100% trichloroacetic acid was added and incubated on ice for 4 h. The centrifuged protein pellet was washed with ice-cold acetone twice and resuspended in 50 μl of SDS-loading buffer; this sample was defined as Sup. A total of 0.5 ml of cell culture was pelleted by centrifugation and resuspended in 50 μl SDS-loading buffer; this sample was defined as cell (Cell). All samples were normalized to the absorbance of the culture in preparation at 600 nm.

For Western blots, protein samples were resolved by SDS-PAGE and transferred onto polyvinylidene fluoride membrane (Millipore). After blocking with 5% milk for 1 h at room temperature, the proteins were probed with primary antibody and horseradish peroxidase–conjugated secondary antibody in Tris-buffered saline with Tween-20 buffer in sequence. The target protein signal was detected by the ECL Plus kit (GE Healthcare) following the manufacturer's protocol.

### Microscopy

For time-lapse analysis, an overnight bacteria culture was diluted 1:100 into LB medium and incubated until an absorbance reached 1.0 at 600 nm at 37 °C. A 1-ml aliquot of the growth was mixed with 100 μl LB supplemented with IPTG to give a final concentration of 1 mM. The mixture was placed on 1% agarose pads and examined over 30 min with a rate of 1 image per 30 s at room temperature. The movie is played at 10 frames per second with Fiji ImageJ. A Nikon Ti2-E inverted microscope with a perfect focus system and a CFI Plan Apo Lambda ×3100 oil Ph3 DM (numerical aperture: 1.4) objective lens were used for imaging. NIS-Elements AR 5.20.00 was used to record and manipulate the images.

For membrane permeability transition, *E. coli* BL21(DE3) (pGEX-6p-1) harboring an empty expression vector pET28a or pET28a-*pseM* were grown in LB with 0.1 mM IPTG to an absorbance of 0.6 at 600 nm. A 1-ml portion of cell cultures was fixed with 4% paraformaldehyde for 20 min. The fixed cells were mock-treated or treated with lysozyme (500 μg/ml) for 1 h in PBS at room temperature. After being washed twice with PBS and incubated with 1% Triton X-100 in PBS for 30 min, the samples were incubated with primary anti-GST mouse antibody in PBS containing 1% bovine serum albumin and 0.01% Triton X-100 overnight at 4 °C. The primary antibody was removed by washing with PBS containing 0.01% Triton X-100 and further incubated with Alexa-Fluor 647–conjugated anti-mouse antibodies in PBS containing 0.01% Triton X-100 for 1 h at room temperature, protected from light. After being washed with PBS containing 0.01% Triton X-100, the cell membrane was further stained with FM1-43 dye, and the resulting cells were analyzed by microscope.

### Protein expression and purification

The His-tag PseM protein purification was performed as described previously (36). *E. coli* BL21(DE3) was transformed with plasmids pET28a-*pseM*. *E. coli* BL21(DE3) (pET28a-*pseM*) was grown at 37 °C in 10 ml LB medium overnight, and then the overnight culture was subcultured with a 1 to 100 dilution into LB medium. The cells were grown at 37 °C, 220 rpm to an absorbance of ∼0.7 at 600 nm, and then the temperature was reduced to 16 °C and expression was induced with 0.5 mM IPTG for another 20 h. The cells were harvested by centrifugation, and the pellet was resuspended in 80 ml buffer A (50 mM Tris–HCl (pH 7.5), 500 mM NaCl, and 1 mM PMSF) with 0.5 mg/ml lysozyme. The cells were disrupted by sonication on ice until the suspension was translucent and centrifuged at 13,400*g* for 25 min. The protein was purified with an AKTA pure protein purification system (GE Healthcare). The Sup was filtered through a 0.45 μm filter and applied to a HisTrap HP column (GE Healthcare) equilibrated with buffer A. After sufficient washing with 10% buffer B (90% buffer A), the column was eluted with a 50-ml linear gradient of 50 to 500 mM imidazole in 10% to 100% buffer B (50 mM Tris–HCl [pH 7.5], 500 mM NaCl, 1 mM PMSF, and 500 mM imidazole). Peak fractions were pooled and checked by SDS-PAGE. The fractions with the desired protein were pooled and desalted with a HiTrap Desalting column (GE Healthcare) by buffer C (50 mM Tris–HCl [pH 7.5], 200 mM NaCl, and 10% glycerol). The purified PseM protein was concentrated and stored at −80 °C before using.

### Co-IP assay

*In vivo* co-IP assay was performed as described previously (47). A 20-ml aliquot of overnight cultures of *P. aeruginosa* transformed by pMMB67HE-PA0990-VSV with either mini-CTX-*pseM*-FLAG or mini-CTX-*hcp1*-FLAG was harvested and lysed by sonication on ice with co-IP lysis buffer (20 mM Tris, 150 mM NaCl, 0.1% Triton X-100, pH 8.0). The lysis Sup was incubated with 50 μl anti-VSV beads (Sigma–Aldrich) for 2 h at 4 °C, and the unbound proteins were sufficiently washed with co-IP washing buffer (20 mM Tris, 500 mM NaCl, 0.1% Triton X-100, pH 8.0). Beads coated with proteins were resolved by SDS-PAGE, and target proteins were detected by Western blot against α-FLAG or α-VSV antibody.

### Muramidase activity of PseM by UPLC–MS

Muramidase activity assays were performed according to a literature method (48). The synthetic peptidoglycans used in this study were synthesized by the Mobashery laboratory earlier (28, 29, 30). The PseM, mutanolysin (Sigma–Aldrich), or AmpDh3 (32) were incubated with the synthetic peptidoglycans or purified *E. coli* sacculus substrate in 20 mM phosphate buffer (pH 8.0), 150 mM NaCl, and 100 μM ZnCl_2_ for 20 h at room temperature. The reactions were stopped by boiling for 5 min, the mixtures were reduced (for reactions with sacculus), and they were flash frozen until UPLC–MS analysis. The UPLC–MS instrumentation and conditions were carried out as reported previously with slight modifications with the LC gradients (49). The reaction mixture was separated and analyzed with a C18 reversed-phase UPLC column (Acquity UPLC HSS T3, 1.8 μm, 2.1 × 150 mm; Waters). A 25-min LC gradient was performed for reactions of compound **4** as follows: held at 5% B for 2 min, ramped to 20% B for 18.9 min, ramped to 5% B for 0.1 min, and held at 5% B for 4 min (A = 0.1% formic acid in water, B = 0.1% formic acid in acetonitrile) at a flow rate of 0.4 ml/min. LC flow during the first 1.5 min was diverted to the waste after confirming that no reaction products were eluted during the period. For reactions with the sacculus, a 35-min LC gradient was performed as follows: held at 0% B for 5 min, ramped to 17% B for 30 min at a flow rate of 0.4 ml/min. LC flow during the first 3.5 min was diverted to the waste after confirming that no reaction products were eluted during the period.

### Protein crystallization and data collection

Purified N-terminal-truncated selenomethionine derivatives or native PseM protein (residues from 28 to 186) were at 27 mg/ml and mixed with an equal volume of reservoir solution, followed by equilibration with 100 μl of the reservoir solution for crystallization at 291 K. Tube-shaped crystals were observed after 2 weeks in the reservoir solution containing 0.1 M Mes monohydrate, pH 7.0, 2.0 M NaCl, and 1% Ben–HCl. The crystals were subsequently soaked in protectant containing mother liquor supplemented with 30% glycerol (v/v) and flash frozen in liquid nitrogen for X-ray diffraction. The diffraction data were collected at the BL19U1 beamline of the National Facility for Protein Science in Shanghai at the Shanghai Synchrotron Radiation Facility and processed with HKL3000 (50) (Table S1). The phase of the structure was solved by single-wavelength anomalous diffraction to under 2.0 Å resolution. Subsequently, the native protein structure was determined and refined using Phenix (Phenix Industrial Consortium) (51), and the model was conducted by the graphics program Coot (STFC Rutherford Appleton Laboratory) (52). The structure has been deposited into the Protein Data Bank (accession code: 9M3L).

### Bacterial competition assay

Bacterial competition assays were performed as described previously (36). Briefly, overnight cultures of relevant *P. aeruginosa* strains and *E. coli* or *Yptb* harboring a gentamicin resistance plasmid pBBR1-MCS5 were mixed 1:1, then 5 μl of each mixture was spotted on a nitrocellulose membrane placed on LB–LS 3% agar. After incubating for 24 h at 37 °C, the cells were resuspended in 1 ml LB and serially diluted, separated on PIA or LB plates containing 25 μg/ml gentamicin, and the final colony-forming unit was determined. The colony-forming units (*E. coli/P. aeruginosa* and *Yptb/P. aeruginosa*) were calculated with appropriate dilution factors.

### Sequence analysis and phylogeny of PseM

The protein sequence of PA0989 was retrieved from the National Center for Biotechnology Information database (accession no.: AAG04378). Briefly, BLASTp searches against GenBank-archived bacterial genomes were conducted to acquire PA0989 homologs by using the following criteria (identities ≥ 50, e value ≤ 0.001, and manual curation was required if necessary). These candidates were included in the subsequent phylogenetic analysis. Multiple sequence alignment was performed for homologous proteins in this repertoire by using MUSCLE (53). Next, the phylogeny was calculated with MEGA, version 11 (54). Moreover, the neighbor-joining method and Jones–Taylor–Thornton model with 1000 bootstrap replicates were conducted to infer their evolutionary relationship.

## Data availability

The data that support the findings of this study are available in the supporting information of this article. Correspondence and requests for materials should be addressed to Dr Haihua Liang.

## Supporting information

This article contains supporting information.

## Conflict of interest

The authors declare that they have no conflicts of interest with the contents of this article.

## Supporting information

Resource data for figures

## References

[1] Layeghifard M., Hwang D.M., Guttman D.S. (2017). Disentangling interactions in the microbiome: a network perspective. Trends Microbiol..

[2] Coyte K.Z., Rakoff-Nahoum S. (2019). Understanding competition and cooperation within the mammalian gut microbiome. Curr. Biol..

[3] Asfahl K.L., Schuster M. (2017). Social interactions in bacterial cell-cell signaling. FEMS Microbiol. Rev..

[4] Kohler T., Buckling A., van Delden C. (2009). Cooperation and virulence of clinical *Pseudomonas aeruginosa* populations. Proc. Natl. Acad. Sci. U. S. A..

[5] Chassaing B., Cascales E. (2018). Antibacterial weapons: targeted destruction in the microbiota. Trends Microbiol..

[6] Meir A., Mace K., Vegunta Y., Williams S.M., Waksman G. (2023). Substrate recruitment mechanism by gram-negative type III, IV, and VI bacterial injectisomes. Trends Microbiol..

[7] Green E.R., Mecsas J. (2016). Bacterial secretion systems: an overview. Microbiol. Spectr..

[8] Costa T.R., Felisberto-Rodrigues C., Meir A., Prevost M.S., Redzej A., Trokter M. (2015). Secretion systems in Gram-negative bacteria: structural and mechanistic insights. Nat. Rev. Microbiol..

[9] Ma L.S., Hachani A., Lin J.S., Filloux A., Lai E.M. (2014). *Agrobacterium tumefaciens* deploys a superfamily of type VI secretion DNase effectors as weapons for interbacterial competition in planta. Cell Host Microbe.

[10] Song L., Pan J., Yang Y., Zhang Z., Cui R., Jia S. (2021). Contact-independent killing mediated by a T6SS effector with intrinsic cell-entry properties. Nat. Commun..

[11] Mariano G., Trunk K., Williams D.J., Monlezun L., Strahl H., Pitt S.J. (2019). A family of Type VI secretion system effector proteins that form ion-selective pores. Nat. Commun..

[12] Stover C.K., Pham X.Q., Erwin A.L., Mizoguchi S.D., Warrener P., Hickey M.J. (2000). Complete genome sequence of *Pseudomonas aeruginosa* PAO1, an opportunistic pathogen. Nature.

[13] Lee J., Zhang L. (2015). The hierarchy quorum sensing network in *Pseudomonas aeruginosa*. Protein Cell.

[14] Moradali M.F., Ghods S., Rehm B.H. (2017). *Pseudomonas aeruginosa* lifestyle: a paradigm for adaptation, survival, and persistence. Front. Cell Infect. Microbiol..

[15] Tashiro Y., Yawata Y., Toyofuku M., Uchiyama H., Nomura N. (2013). Interspecies interaction between *Pseudomonas aeruginosa* and other microorganisms. Microbe Environ..

[16] Ma Q., Zhai Y., Schneider J.C., Ramseier T.M., Saier M.H. (2003). Protein secretion systems of *Pseudomonas aeruginosa* and *P fluorescens*. Biochim. Biophys. Acta.

[17] Jiang F., Waterfield N.R., Yang J., Yang G., Jin Q. (2014). A *Pseudomonas aeruginosa* type VI secretion phospholipase D effector targets both prokaryotic and eukaryotic cells. Cell Host Microbe.

[18] Russell A.B., LeRoux M., Hathazi K., Agnello D.M., Ishikawa T., Wiggins P.A. (2013). Diverse type VI secretion phospholipases are functionally plastic antibacterial effectors. Nature.

[19] Hood R.D., Singh P., Hsu F., Guvener T., Carl M.A., Trinidad R.R. (2010). A type VI secretion system of *Pseudomonas aeruginosa* targets a toxin to bacteria. Cell Host Microbe.

[20] Russell A.B., Hood R.D., Bui N.K., LeRoux M., Vollmer W., Mougous J.D. (2011). Type VI secretion delivers bacteriolytic effectors to target cells. Nature.

[21] Teufel F., Almagro Armenteros J.J., Johansen A.R., Gislason M.H., Pihl S.I., Tsirigos K.D. (2022). SignalP 6.0 predicts all five types of signal peptides using protein language models. Nat. Biotechnol..

[22] Andresen L., Martinez-Burgo Y., Nilsson Zangelin J., Rizvanovic A., Holmqvist E. (2020). The small toxic *salmonella* protein TimP targets the cytoplasmic membrane and is repressed by the small RNA TimR. mBio.

[23] Zhang D., Iyer L.M., Aravind L. (2011). A novel immunity system for bacterial nucleic acid degrading toxins and its recruitment in various eukaryotic and DNA viral systems. Nucleic Acids Res..

[24] Goodman A.L., Kulasekara B., Rietsch A., Boyd D., Smith R.S., Lory S. (2004). A signaling network reciprocally regulates genes associated with acute infection and chronic persistence in *Pseudomonas aeruginosa*. Dev. Cell.

[25] Records A.R., Gross D.C. (2010). Sensor kinases RetS and LadS regulate *Pseudomonas syringae* type VI secretion and virulence factors. J. Bacteriol..

[26] Zuker M. (2003). Mfold web server for nucleic acid folding and hybridization prediction. Nucleic Acids Res..

[27] Mougous J.D., Cuff M.E., Raunser S., Shen A., Zhou M., Gifford C.A. (2006). A virulence locus of *Pseudomonas aeruginosa* encodes a protein secretion apparatus. Science.

[28] Lee M., Hesek D., Lastochkin E., Dik D.A., Boggess B., Mobashery S. (2017). Deciphering the nature of enzymatic modifications of bacterial cell walls. Chembiochem.

[29] Martinez-Caballero S., Lee M., Artola-Recolons C., Carrasco-Lopez C., Hesek D., Spink E. (2013). Reaction products and the X-ray structure of AmpDh2, a virulence determinant of *Pseudomonas aeruginosa*. J. Am. Chem. Soc..

[30] Lee M., Hesek D., Shah I.M., Oliver A.G., Dworkin J., Mobashery S. (2010). Synthetic peptidoglycan motifs for germination of bacterial spores. Chembiochem.

[31] Byun B., Mahasenan K.V., Dik D.A., Marous D.R., Speri E., Kumarasiri M. (2018). Mechanism of the *Escherichia coli* MltE lytic transglycosylase, the cell-wall-penetrating enzyme for type VI secretion system assembly. Sci. Rep..

[32] Zhang W., Lee M., Hesek D., Lastochkin E., Boggess B., Mobashery S. (2013). Reactions of the three AmpD enzymes of *Pseudomonas aeruginosa*. J. Am. Chem. Soc..

[33] Holm L., Laiho A., Toronen P., Salgado M. (2023). DALI shines a light on remote homologs: one hundred discoveries. Protein Sci..

[34] Eberhardt J., Santos-Martins D., Tillack A.F., Forli S. (2021). AutoDock vina 1.2.0: new docking methods, expanded force field, and python bindings. J. Chem. Inf. Model.

[35] Juan C., Moya B., Perez J.L., Oliver A. (2006). Stepwise upregulation of the *Pseudomonas aeruginosa* chromosomal cephalosporinase conferring high-level beta-lactam resistance involves three AmpD homologues. Antimicrob. Agents Chemother..

[36] Wang T., Hu Z., Du X., Shi Y., Dang J., Lee M. (2020). A type VI secretion system delivers a cell wall amidase to target bacterial competitors. Mol. Microbiol..

[37] Alcorlo M., Dik D.A., De Benedetti S., Mahasenan K.V., Lee M., Dominguez-Gil T. (2019). Structural basis of denuded glycan recognition by SPOR domains in bacterial cell division. Nat. Commun..

[38] Aoki S.K., Diner E.J., de Roodenbeke C.T., Burgess B.R., Poole S.J., Braaten B.A. (2010). A widespread family of polymorphic contact-dependent toxin delivery systems in bacteria. Nature.

[39] Song L., Xu L., Wu T., Shi Z., Kareem H.A., Wang Z. (2024). Trojan horselike T6SS effector TepC mediates both interference competition and exploitative competition. ISME J..

[40] Anderson M.C., Vonaesch P., Saffarian A., Marteyn B.S., Sansonetti P.J. (2017). *Shigella sonnei* encodes a functional T6SS used for interbacterial competition and niche occupancy. Cell Host Microbe.

[41] Tsirigotaki A., De Geyter J., Sostaric N., Economou A., Karamanou S. (2017). Protein export through the bacterial sec pathway. Nat. Rev. Microbiol..

[42] Matsuda S., Okada R., Tandhavanant S., Hiyoshi H., Gotoh K., Iida T. (2019). Export of a *Vibrio parahaemolyticus* toxin by the sec and type III secretion machineries in tandem. Nat. Microbiol..

[43] Cianfanelli F.R., Monlezun L., Coulthurst S.J. (2016). Aim, load, fire: the type VI secretion system, a bacterial nanoweapon. Trends Microbiol..

[44] Zoued A., Brunet Y.R., Durand E., Aschtgen M.S., Logger L., Douzi B. (2014). Architecture and assembly of the type VI secretion system. Biochim. Biophys. Acta.

[45] Wang T., Si M., Song Y., Zhu W., Gao F., Wang Y. (2015). Type VI secretion system transports Zn^2+^ to combat multiple stresses and host immunity. PLoS Pathog..

[46] Imperi F., Ciccosanti F., Perdomo A.B., Tiburzi F., Mancone C., Alonzi T. (2009). Analysis of the periplasmic proteome of *Pseudomonas aeruginosa*, a metabolically versatile opportunistic pathogen. Proteomics.

[47] Han Y., Wang T., Chen G., Pu Q., Liu Q., Zhang Y. (2019). A *Pseudomonas aeruginosa* type VI secretion system regulated by CueR facilitates copper acquisition. PLoS Pathog..

[48] Lee M., Artola-Recolons C., Carrasco-Lopez C., Martinez-Caballero S., Hesek D., Spink E. (2013). Cell-wall remodeling by the zinc-protease AmpDh3 from *Pseudomonas aeruginosa*. J. Am. Chem. Soc..

[49] Kim C., Lee M., Birhanu B.T., Hesek D., Chang M., Mobashery S. (2023). Synthesis of muramyl-delta-lactam in spore peptidoglycan of *Clostridioides difficile*. Chembiochem.

[50] Minor W., Cymborowski M., Otwinowski Z., Chruszcz M. (2006). HKL-3000: the integration of data reduction and structure solution--from diffraction images to an initial model in minutes. Acta Crystallogr. D Biol. Crystallogr..

[51] Adams P.D., Grosse-Kunstleve R.W., Hung L.W., Ioerger T.R., McCoy A.J., Moriarty N.W. (2002). PHENIX: building new software for automated crystallographic structure determination. Acta Crystallogr. D Biol. Crystallogr..

[52] Emsley P., Cowtan K. (2004). Coot: model-building tools for molecular graphics. Acta Crystallogr. D Biol. Crystallogr..

[53] Edgar R.C. (2004). MUSCLE: a multiple sequence alignment method with reduced time and space complexity. BMC Bioinformatics.

[54] Tamura K., Stecher G., Kumar S. (2021). MEGA11: molecular evolutionary genetics analysis version 11. Mol. Biol. Evol..

