# Supplementary material for: A Pseudomonas aeruginosa endolytic muramidase targets cell-wall peptidoglycan in bacterial competition
Source: J Biol Chem. 2025 Aug 28;301(10):110642. doi: 10.1016/j.jbc.2025.110642 (PMC12494544; doi:10.1016/j.jbc.2025.110642)
Supplement: Supporting Information [file mmc2.pdf]

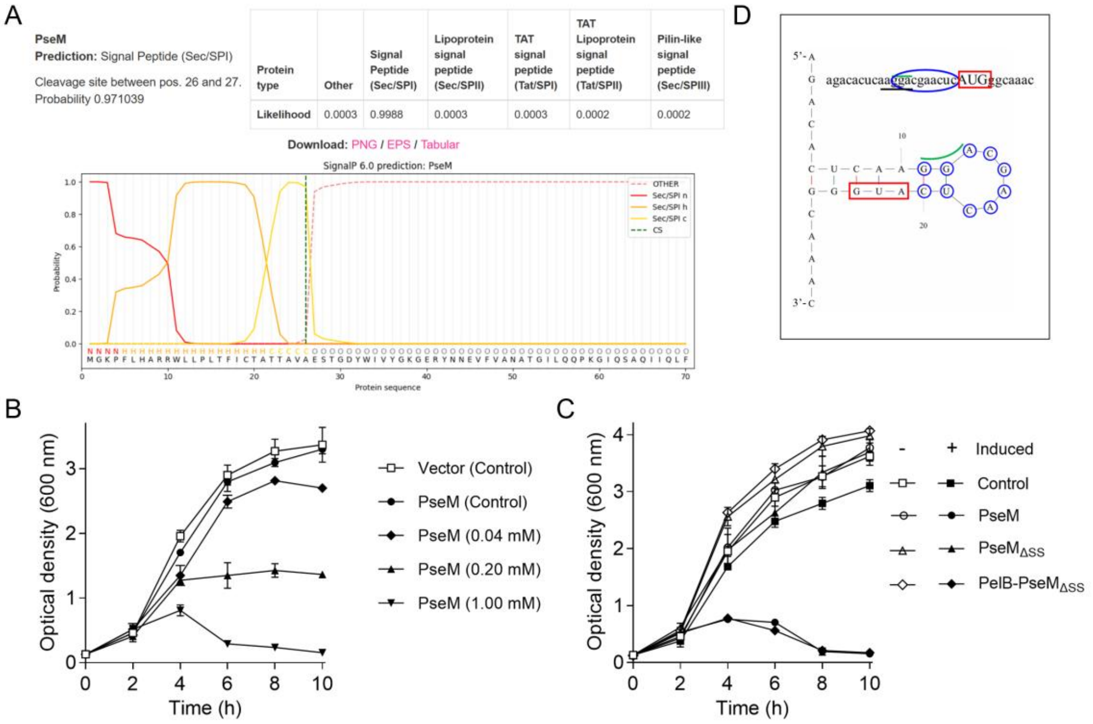

Fig. S1. Overexpression of PseM suppresses bacterial growth. (A) Signal peptide prediction of PseM. The protein sequence was uploaded to online server SignalP-6.0 (https://services.healthtech.dtu.dk/services/SignalP-6.0/), the result suggested that the N-terminus of PseM contains a signal peptide (residues 10-21, Sec/SPI, orange curve, probability 99.88%) with cleavage site between residues 26 and 27 probability 97.10%). OTHER (red dotted line) represent no signal peptide, Sec/SPI n (red line), Sec/SPI h (deep yellow line) and Sec/SPI c (yellow line) represent the n-terminal region, center hydrophobic region and c-terminal region of Sec signal peptide respectively, CS (green dotted line) represent the cleavage site of the signal peptide. **(B)** Growth curves of *E. coli* BL21(DE3) pLysS producing PseM (with IPTG concentrations indicated in parentheses) cultured in LB broth. The control group represents cultures without IPTG induction. **(C)** Growth curves of *E. coli* BL21 (DE3) pLysS strains harboring an empty vector (Control), full-length PseM (PseM), PseM lacking the signal peptide (PseM$_{ΔSS}$), or PelB-fused PseM$_{ΔSS}$ (PelB-PseM$_{ΔSS}$) in LB broth under uninduced and induced conditions with 1.0 mM IPTG. (*B, C*) Error bars indicate mean ± SD (n = 2). **(D)** Mfold-predicted RsmA binding sites within the *pseM* promoter region. Key features: blue ovals/circles, putative RsmA binding motifs; green line, core GGA sequence; red box, translation start codon (ATG); black underline - predicted ribosome binding site (RBS).

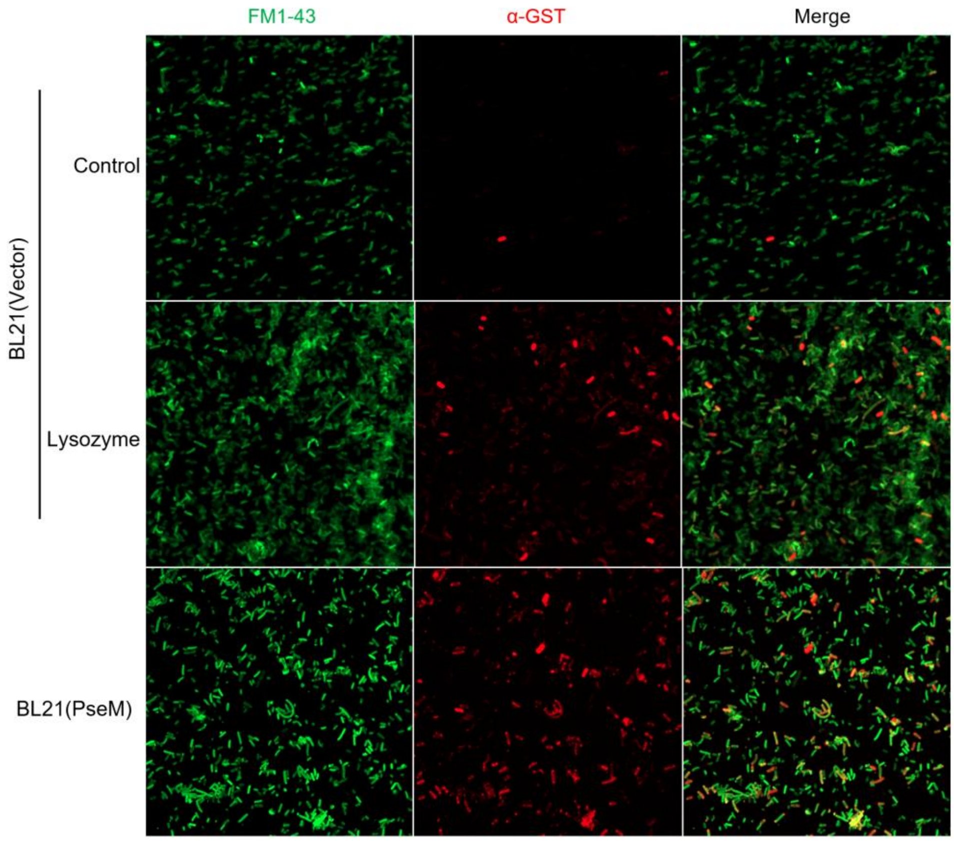

Fig. S2. PseM induce bacterial cell-wall destruction. Full fields of micrographs are related to Figure. 3A. *E. coli* BL21 (DE3) harboring an empty expression vector pET28a (BL21(Vector); producing glutathione S-transferase (GST)) or pET28a-*pseM* (BL21(PseM)) were grown in LB supplemented with 0.1 mM IPTG to OD_{600}=0.6. Then the cells were fixed, mock-treated or treated with lysozyme, and exposed to mouse anti-GST and Alexa-Fluor 647-conjugated anti-mouse antibodies. The cells membrane was further staining with FM1-43 dye, and the resulting cells were analyzed by a Nikon Ti2-E inverted microscope. Data are representative of three independent replicates.

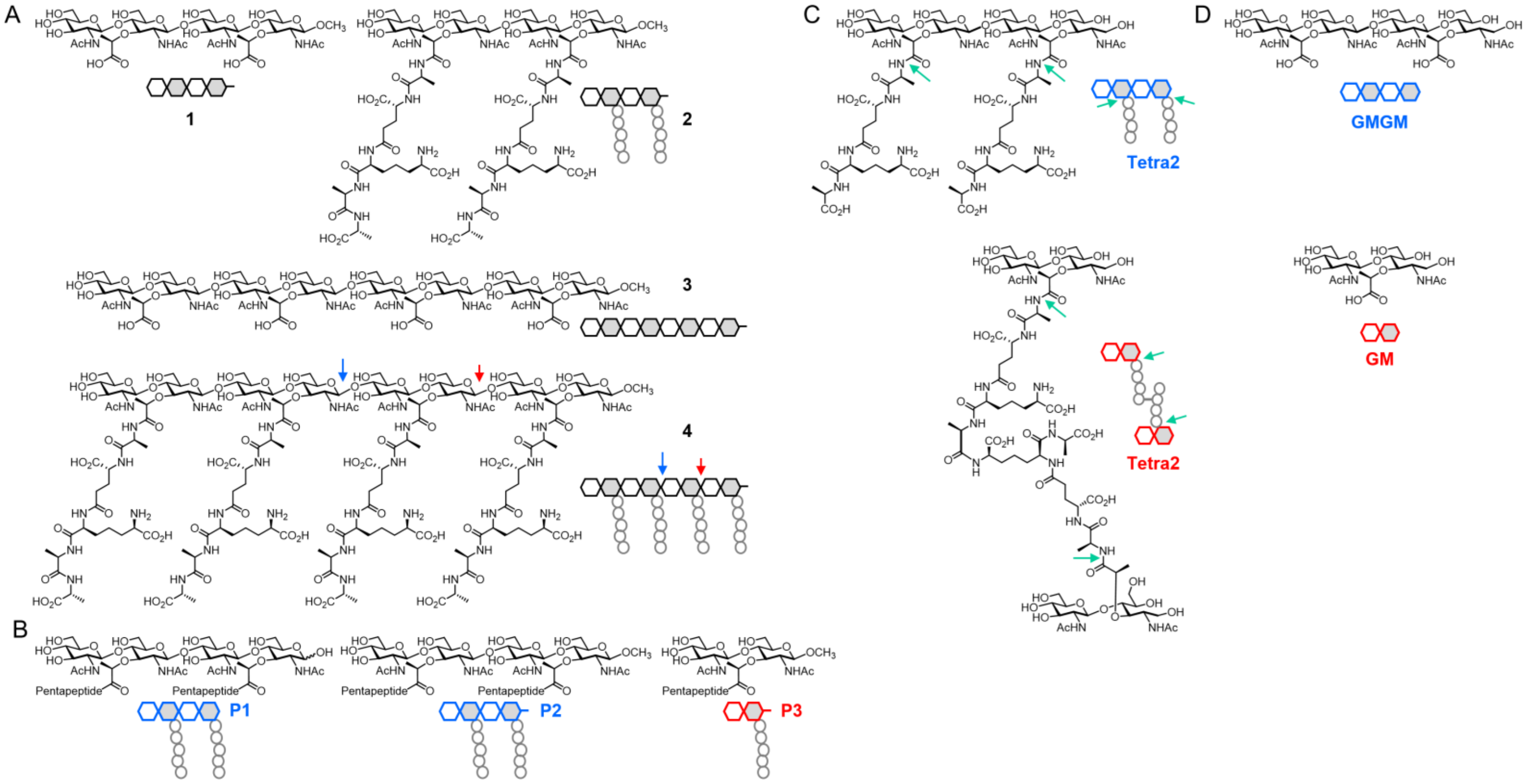

Fig. S3. Chemical structures of synthetic peptidoglycans used in this study and reaction products. (A) Four synthetic peptidoglycans (**1-4**) and **(B)** PseM reaction products from the reaction with **4**. **(C)** Reaction products with sacculus catalyzed by PseM and by mutanolysin. Tetrapeptide-containing products are depicted as representative examples. **(D)** AmpDh3 amidase reaction products of sacculus predigested by PseM or by mutanolysin to simplify reaction outcome. Structure shown in blue were uniquely found in the reaction of PseM with the sacculus and those in red were found in both mutanolysin and PseM reactions. Blue and red arrow in compound **4** in panel A indicate the major and minor cleavage sites of PseM and green arrows in panel C indicate the cleavage sites of amidase AmpDh3.

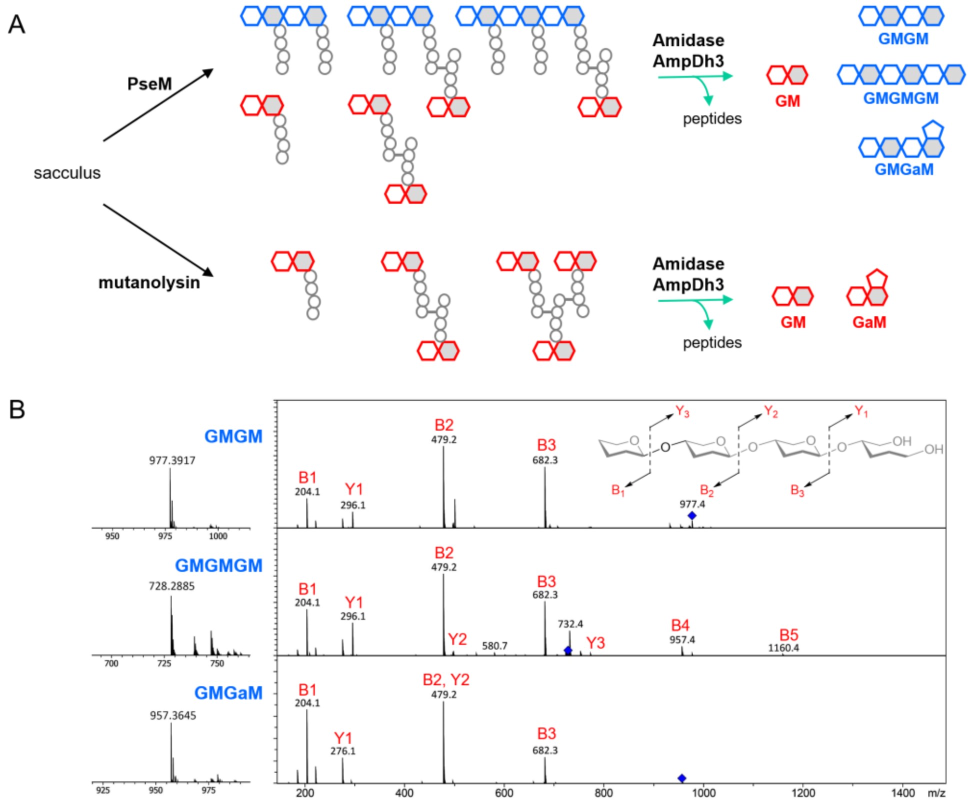

Fig. S4. (A) Reactions of PseM (top panel) with the sacculus, followed by amidase AmpDh3. Reactions of mutanolysin (bottom panel) with the sacculus, followed by the amidase AmpDh3. **(B)** The mass spectra of endolytic reaction products (left panel, with +1 charge state as $[M+H]^+$ except 728 (with +2 charge state as $[M+2H]^{2+}$)) and corresponding collision-induced dissociation mass spectra (right panel). Fragmentation naming convention for hexose is given in the top right corner of panel B.

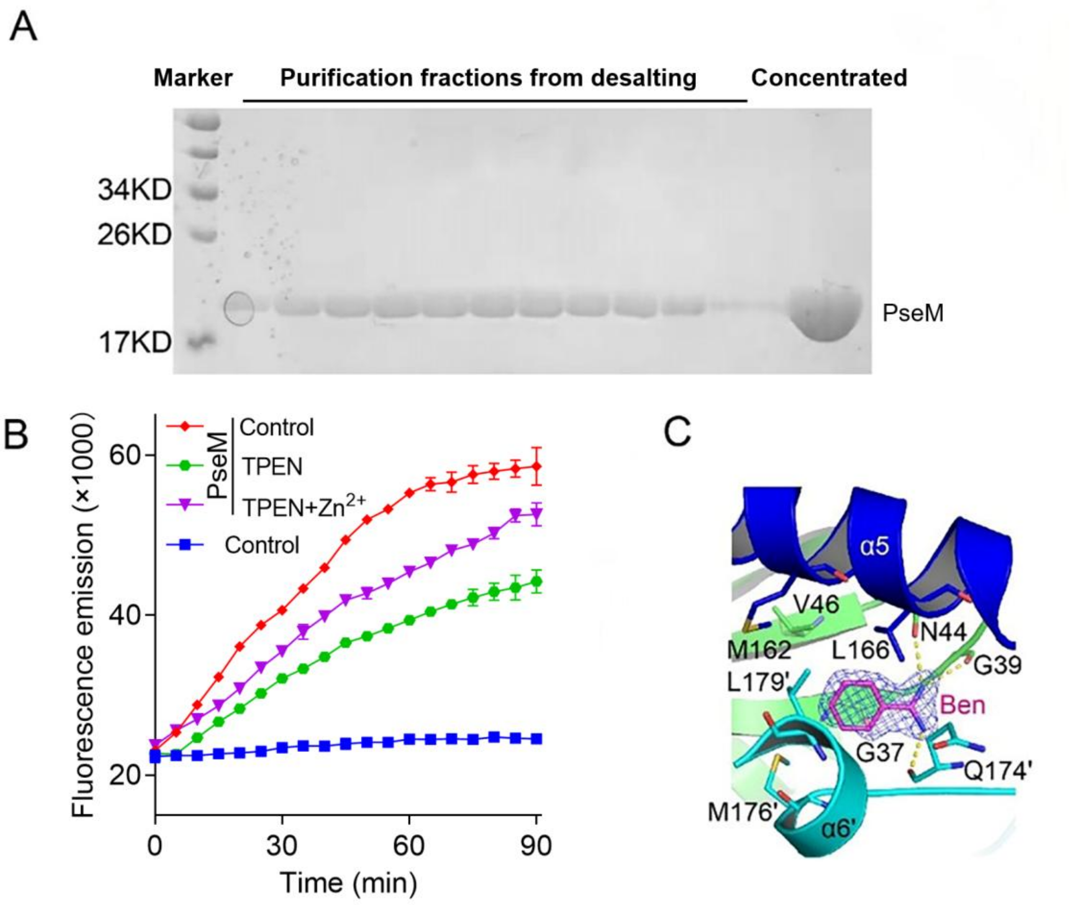

Fig. S5. Structural analysis of PseM. (A) PseM purification analysis by SDS-PAGE. **(B)** Zinc-dependent enzymatic activity. Lytic activity against peptidoglycan was quantified fluorometrically using the EnzChek Lysozyme Assay Kit. Experimental groups: PseM alone, PseM + 10 μM zinc chelator TPEN (N, N, N', N'-Tetrakis (2-pyridylmethyl) ethylenediamine), and PseM + TPEN + 10 μM Zn^{2+}. Data shown as mean ± SD (n=3 biological replicates). **(C)** Ligand-binding interface analysis. Fo-fc omit electron density map contoured at 3.0 σ around the benzamidine (Ben) molecule. The residues that are involved in the interactions are shown in capped sticks and labeled.

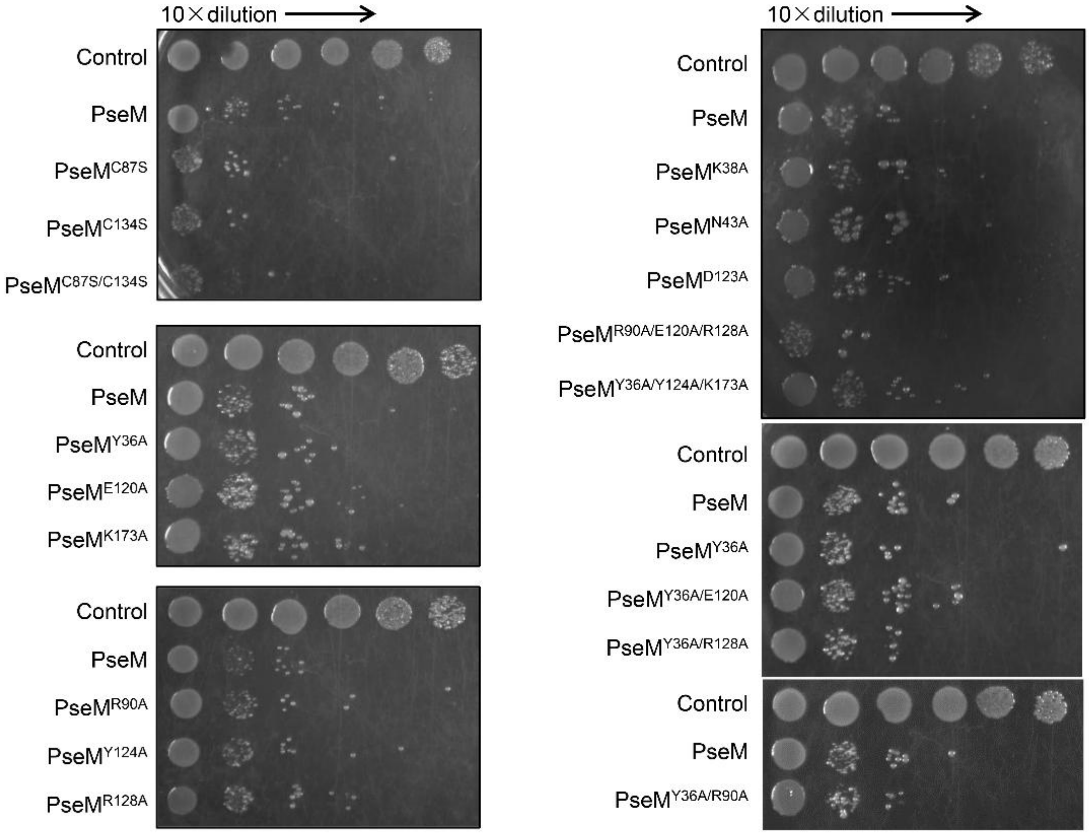

Fig. S6. *In vivo* functional analysis of PseM site-directed mutagenesis variants. Growth of *E. coli* strain BL21 (DE3) pLysS harboring an empty vector (Control), wild-type PseM (PseM) or specified site-mutated PseM variants grown on LB agar plates supplemented with 1.0 mM IPTG at 37°C. Results shown are representative of three independent biological replicates.

Table S1. Data collection and refinement statistics.

	PseM (Selenomethionine-SAD, Se-SAD)	PseM (Native protein)
**Data collection**		
Space group	$P4_32_12$	$P4_32_12$
Cell dimensions		
a, b, c (Å)	97.86, 97.86, 92.31	97.73, 97.73, 92.15
α, β, γ (°)	90, 90, 90	90, 90, 90
Wavelength (Å)	0.9785	0.9785
Resolution (Å)	50.0-2.00 (2.07-2.00)	50.0-2.00 (2.07-2.00)
R_{merge} (%)	10.2(151.2)	10.3(150.9)
$I/\sigma I$	39.2(3.0)	34.0(2.0)
Completeness (%)	100(100)	100(100)
Redundancy	24.2(17.1)	25.2(20.4)
CC1/2 (%)	98.6(85.8)	96.0(69.4)
**Refinement**		
Resolution (Å)	50-2.00	50-2.00
No. reflections	30525	29268
R_{work}/R_{free}	0.208/0.234	0.223/0.250
No. atoms		
Protein	2525	2550
Water	262	198
Ligand/ion	2	29
B-factors		
Protein	27.73	30.93
Water	33.59	32.71
Ligand/ion	25.70	28.90
R.m.s deviations		
Bond lengths (Å)	0.007	0.013
Bond angles (°)	0.824	1.045

Table S2. Bacterial strains and plasmids used in this study.

Strain or plasmid	Relevant characteristics	Source
E. coli		
DH5α	*F⁻ φ80lacZ ΔM15 Δ(lacZYA-argF) U169 recA1 endA1 hsdR17(rk⁻, mk⁺) phoA supE44 thi-1 gyrA96 relA1 tonA*	Stratagene
BL21(DE3)	F^- *ompT hsdS*$_B$ (r_B^- m_B^-) *gal dcm* (DE3)	Invitrogen
BL21(DE3) plysS	F^- *ompT hsdS*$_B$ (r_B^- m_B^-) *gal dcm* (DE3) plysS, Cm^r	Invitrogen
Y. pseudotuberculosis		
Yptb	Wild type *Yersinia pseudotuberculosis*	(1)
P. aeruginosa		
PAO1	Wild type *Pseudomonas aeruginosa* PAO1	This lab
Δ*retS*	*retS* deletion mutant of PAO1	This lab
Δ*retS*Δ*clpV1*	*retS/clpV1* deletion mutant of PAO1	This lab
Δ*retS*Δ*clpV2*	*retS/clpV2* deletion mutant of PAO1	This lab
Δ*retS*Δ*clpV3*	*retS/clpV3* deletion mutant of PAO1	This lab
Δ*retS*Δ*clpV123*	*retS/clpV1/clpV2/clpV3* deletion mutant of PAO1	This lab
Δ*retS*Δ*hxcR*	*retS/hxcR* deletion mutant of PAO1	This lab
Δ*retS*Δ*pseM*	*retS/pseM* deletion mutant of PAO1	This study
Δ*rsmA*	*rsmA* deletion mutant of PAO1	This lab
**Plasmids**		
pEX18Ap	Gene replacement vector with multiple-cloning site from pUC18; Ap^r	(2)
pEX18Ap-*pseM*	*pseM* deletion plasmid, pEX18Ap with upstream and downstream regions of *pseM*	This study
pEX18Ap-*hxcR*	*hxcR* deletion plasmid, pEX18Ap with upstream and downstream regions of *hxcR*	This study
pAK1900	*E. coli*-*P. aeruginosa* shuttle cloning vector carrying p*lac* upstream of MCS; Ap^r, Cb^r	(3)
pAK-*retS*	pAK1900 with the entire *retS* gene	This Lab
pAK-*rsmA*	pAK1900 with the entire *rsmA* gene	This Lab
pAK-*pseM*	pAK1900 with the entire *pseM* gene	This study
pAK-$pseM^M$	pAK1900 with the entire $pseM^M$ gene	This study
pET28a-*pseM*	The entire gene of *pseM* cloned into pET28a vector	This study
pET28a-$pseM_{\Delta SS}$	Protein expression construct, *pseM* lacking the signal peptide cloned into pET28a vector	This study
pET28a-$pseM^M$	Protein expression construct, $pseM^{Y36A/R90A/E120A/R128A}$ cloned into pET28a vector	This study
pET22b	T7 *lac* promoter-operator, carrying an N-terminal PelB signal sequence, Ap^r	Novagen
pET22b-*pseM*	The entire gene of *pseM* cloned into pET22b vector	This study
pET22b-$pseM_{\Delta SS}$	Protein expression construct, *pseM* lacking the signal sequence cloned into pET22b vector	This study
pET-Duet-1	Dual T7 *lac* promoter-operator, N-terminal His tag, Ap^r	Novagen
pETDuet-*pseM*	Protein expression construct, the entire gene of *pseM*	This study

	cloned into the MCS1 of pET-Duet-1 vector	
pETDuet-*pseM-PA0990*	Protein expression construct, the entire gene of *pseM* and P*A0990* cloned into the MCS1 and MCS2 of pET-Duet-1 vector, respectively	This study
pBBR1-MCS5	Broad-Host-Range vector, Gm^r	(4)
pMMB67HE-*PA0990-VSV*	pMMB67HE containing the entire *PA0990* gene and the VSV sequence	This Study
mini-CTX-*lacZ*-Flag	Integration plasmid, mini-CTX-*lacZ* containing the 3x *flag* sequence, Tc^r	This Lab (5)
Mini-CTX-*hcp1*-Flag	Mini-CTX-*lacZ* containing the entire *hcp1* gene and the 3x *flag* sequence	This Lab
mini-CTX-*pseM*-Flag	mini-CTX-*lacZ* containing the entire *pseM* gene	This Study
mini-CTX-$pseM_{\Delta SS}$-Flag	mini-CTX-*lacZ* containing the entire p$seM_{\Delta SS}$ gene	This Study

Table S3. Primers used in this study.

**Primer**	**Sequence (5'→3')** [a]	**Application**
pEX-*pseM*-Up-S	ATTggatccCAAGTTTGGCGGTATGACC	Constructing
pEX-*PseM*-Up-A	ATTctcgagGATCTGGATGCGATGCTC	*pseM* deletion
pEX-*pseM*-Down-S	TAActcgagCGAGCGTGAAGGAATGGT	mutant
pEX-*pseM*-Down-A	TATaagcttCCAATTCGGTTGAGATACTGAT	
pAK-*pseM*-S	TTTaagcttCCCCCCGACCTCATGGA	Complemented
pAK-*pseM*-A	ATTggatccCTACTCGTTGCCGAGCAT	plasmid
pET-*pseM*-S	ATAccatggTGGGCAAACCATTCCTTCA	Protein cloning
pET-*pseM*$_{\Delta SS}$-S	ATTccatggATGACTGGATCGTCTAC	
pET-*pseM*-A	ATActcgagCTCGTTGCCGAGCATCGC	
pET-*pseM*^{Y36A}-S	GACTACTGGATCGTCGCCGGCAAGGGCGAACG	For *pseM*
pET-*pseM*^{Y36A}-A	CGTTCGCCCTTGCCGGCGACGATCCAGTAGTC	Mutagenesis
pET-*pseM*^{R90A}-S	TTCAAGTGCAAGGAGGCCAGGGTTCGCTTCGA	
pET-*pseM*^{R90A}-A	TCGAAGCGAACCCTGGCCTCCTTGCACTTGAA	
pET-*pseM*^{E120A}-S	GCTGGATCGACCCGGCGAAATACGATTATTG	
pET-*pseM*^{E120A}-A	CAATAATCGTATTTCGCCGGGTCGATCCAGC	
pET-*pseM*^{R128A}-S	GATTATTGGCTGCAAGCCTCGTTCGCCTTCGT	
pET-*pseM*^{R128A}-A	ACGAAGGCGAACGAGGCTTGCAGCCAATAATC	
pET-*pseM*^{C87A}-S	ACCCTGCGCTCCTTGCTCTTGAACTGGACCTCG	
pET-*pseM*^{C87A}-A	CGAGGTCCAGTTCAAGAGCAAGGAGCGCAGGGT	
pET-*pseM*^{C134A}-S	CGGATGGCCGGGGCGCTGACGAAGGCGAACGAG	
pET-*pseM*^{C134A}-A	CTCGTTCGCCTTCGTCAGCGCCCCGGCCATCCG	
pET-*pseM*^{k38A}-S	TGGATCGTCTACGGCGCCGGCGAACGCTACAA	
pET-*pseM*^{K38A}-A	TTGTAGCGTTCGCCGGCGCCGTAGACGATCCA	
pET-*pseM*^{N43A}-S	AAGGGCGAACGCTACGCCAACGAGGTATTCGT	
pET-*pseM*^{N43A}-A	ACGAATACCTCGTTGGCGTAGCGTTCGCCCTT	
pET-*pseM*^{D123A}-S	GACCCGGAGAAATACGCCTATTGGCTGCAACG	
pET-*pseM*^{D123A}-A	CGTTGCAGCCAATAGGCGTATTTCTCCGGGTC	
pET-*pseM*^{K173A}-S	GGCGTCCAGGCCAAGGCACAGACCATGCGCGA	
pET-*pseM*^{K173A}-A	TCGCGCATGGTCTGTGCCTTGGCCTGGACGCC	
pET-Duet-*pseM*-A	ATAaagcttTCAgtgatgatgatgatgatgCTCGTTGCCGAGCATCGC	Protein cloning
	GGCcatatGCCATCGGTCAGCGATCC	
pET-Duet-*PA0990*-S	ATActcgagTCAgtgatgatgatgatgatgGTCGAAAAGCGCCTCTGG	
pET-Duet-*PA0990*-A	C	
PA0990-VSV-S	ATAggtaccATGCCATCGGTCAGCGATCC	PA0990-VSV
PA0990-VSV-A	ATAaagcttTCActtaccgagacggttcatctcgatatcggtgtaGTCGAAAAGCGCCTCTGGC	expression
mini-CTX-*pseM*-Flag-S	ATTggtaccCCCCCCGACCTCATGGA	Western blot
mini-CTX-*pseM*-Flag-A	AATaagcttCTCGTTGCCGAGCATCGC	

[a] Restriction sites displayed in lowercase.

Movie S1. Time-lapse microscopy of *E. coli* strain BL21 (DE3) pLysS harboring an empty vector cultured in LB agar pad supplemented with 1.0 mM IPTG. The growth was imaged over 30 minutes with a rate of 1 image per 30 seconds by a Nikon Ti2-E inverted microscope. The movie was played at a rate of 10 frames per second.

Movie S2. Time-lapse microscopy of *E. coli* strain BL21 (DE3) pLysS harboring an PseM expression vector pET28a-*PseM* cultured in LB agar pad supplemented with 1.0 mM IPTG. The growth was imaged over 30 minutes with a rate of 1 image per 30 seconds by a Nikon Ti2-E inverted microscope. The movie was played at a rate of 10 frames per second.

Supplementary References:

1. Song, Y., Xiao, X., Li, C., Wang, T., Zhao, R., Zhang, W., Zhang, L., Wang, Y., and Shen, X. (2015) The dual transcriptional regulator RovM regulates the expression of AR3- and T6SS4-dependent acid survival systems in response to nutritional status in *Yersinia pseudotuberculosis*. *Environ Microbiol* **17**, 4631-4645
2. Hoang, T. T., Karkhoff-Schweizer, R. R., Kutchma, A. J., and Schweizer, H. P. (1998) A broad-host-range Flp-FRT recombination system for site-specific excision of chromosomally-located DNA sequences: application for isolation of unmarked *Pseudomonas aeruginosa* mutants. *Gene* **212**, 77-86
3. Poole, K., Neshat, S., Krebes, K., and Heinrichs, D. E. (1993) Cloning and nucleotide sequence analysis of the ferripyoverdine receptor gene *fpvA* of *Pseudomonas aeruginosa*. *J Bacteriol* **175**, 4597-4604
4. Kovach, M. E., Elzer, P. H., Hill, D. S., Robertson, G. T., Farris, M. A., Roop, R. M., 2nd, and Peterson, K. M. (1995) Four new derivatives of the broad-host-range cloning vector pBBR1MCS, carrying different antibiotic-resistance cassettes. *Gene* **166**, 175-176
5. Hoang, T. T., Kutchma, A. J., Becher, A., and Schweizer, H. P. (2000) Integration-proficient plasmids for *Pseudomonas aeruginosa*: site-specific integration and use for engineering of reporter and expression strains. *Plasmid* **43**, 59-72
